# Relationships between drought, heat and air humidity responses revealed by transcriptome-metabolome co-analysis

**DOI:** 10.1186/s12870-017-1062-y

**Published:** 2017-07-10

**Authors:** Elisabeth Georgii, Ming Jin, Jin Zhao, Basem Kanawati, Philippe Schmitt-Kopplin, Andreas Albert, J. Barbro Winkler, Anton R. Schäffner

**Affiliations:** 1 Helmholtz Zentrum München, Department of Environmental Sciences, Institute of Biochemical Plant Pathology, Ingolstädter Landstr. 1, 85764 Neuherberg, Germany; 2 Helmholtz Zentrum München, Department of Environmental Sciences, Research Unit Analytical Biogeochemistry, Ingolstädter Landstr, 1, 85764 Neuherberg, Germany; 3 Helmholtz Zentrum München, Department of Environmental Sciences, Research Unit Environmental Simulation, Ingolstädter Landstr, 1, 85764 Neuherberg, Germany

**Keywords:** Abiotic stress systems biology, Transcriptome-metabolome relationships, Molecular interaction effects, Stress decomposition, Air humidity effect, Aquaporins

## Abstract

**Background:**

Elevated temperature and reduced water availability are frequently linked abiotic stresses that may provoke distinct as well as interacting molecular responses. Based on non-targeted metabolomic and transcriptomic measurements from *Arabidopsis* rosettes, this study aims at a systematic elucidation of relevant components in different drought and heat scenarios as well as relationships between molecular players of stress response.

**Results:**

In combined drought-heat stress, the majority of single stress responses are maintained. However, interaction effects between drought and heat can be discovered as well; these relate to protein folding, flavonoid biosynthesis and growth inhibition, which are enhanced, reduced or specifically induced in combined stress, respectively. Heat stress experiments with and without supplementation of air humidity for maintenance of vapor pressure deficit suggest that decreased relative air humidity due to elevated temperature is an important component of heat stress, specifically being responsible for hormone-related responses to water deprivation. Remarkably, this “dry air effect” is the primary trigger of the metabolomic response to heat. In contrast, the transcriptomic response has a substantial temperature component exceeding the dry air component and including up-regulation of many transcription factors and protein folding-related genes. Data level integration independent of prior knowledge on pathways and condition labels reveals shared drought and heat responses between transcriptome and metabolome, biomarker candidates and co-regulation between genes and metabolic compounds, suggesting novel players in abiotic stress response pathways.

**Conclusions:**

Drought and heat stress interact both at transcript and at metabolite response level. A comprehensive, non-targeted view of this interaction as well as non-interacting processes is important to be taken into account when improving tolerance to abiotic stresses in breeding programs. Transcriptome and metabolome may respond with different extent to individual stress components. Their contrasting behavior in response to temperature stress highlights that the protein folding machinery effectively shields the metabolism from stress. Disentangling the complex relationships between transcriptome and metabolome in response to stress is an enormous challenge. As demonstrated by case studies with supporting evidence from additional data, the large dataset provided in this study may assist in determining linked genetic and metabolic features as candidates for future mechanistic analyses.

**Electronic supplementary material:**

The online version of this article (doi:10.1186/s12870-017-1062-y) contains supplementary material, which is available to authorized users.

## Background

Abiotic stresses and stress combinations impact agricultural production by reducing plant growth and yield [1]. A molecular understanding of plant responses and adaptations to various abiotic stress scenarios is crucial for developing breeding and farming strategies that can sustain crop output under changing climate conditions. Drought and heat phases occur frequently – both in separate and in overlapping scenarios. The physiological consequences for plant leaves are partly similar, partly contrary between drought and heat [1]. Therefore, studying drought and heat interactions at the molecular level is relevant for identifying stress components and relationships between stress types. These insights will in turn help adjusting strategies to improve plant stress tolerance to different geographic and climatic situations and making appropriate trade-offs regarding benefits and disadvantages of specific genetic traits for typical stress profiles.

A rise in temperature leads to a decreased relative humidity of the ambient air and an increased vapor pressure deficit (VPD). Some previous heat stress experiments controlled the relative air humidity [2, 3]. Instead of sticking to a specific relative air humidity setting, we make a systematic comparison between an increase in temperature at constant absolute air humidity, hence with increased VPD, and higher temperature with compensatory air humidity supplementation to maintain the same VPD as before the heat stress. This allows for a clear separation of solely temperature-dependent and air humidity-dependent components of heat stress. Furthermore, both heat stress air humidity settings are also combined with preceding drought stress to study how relative air humidity effects under heat are modulated by drought.

Our study uses the model plant *Arabidopsis thaliana*, for which comprehensive knowledge about gene function, regulation and expression as well as metabolic pathways is available [4]. Numerous studies have investigated transcriptomic responses of *A. thaliana* plants to abiotic stress scenarios [2, 5–8] as well as metabolomic stress responses [9, 10]. However, even for this well-studied species the metabolome content of cells, the interplay between transcriptome and metabolome and the mechanistic processes underlying single and combined stress responses are still largely unknown. Here, we characterize responses to drought, heat and combined stress scenarios by non-targeted metabolome profiles of 103 samples in parallel to whole-genome transcriptomics of 135 samples, extending the scope beyond predefined metabolites in contrast to targeted approaches used by the vast majority of previous studies [2, 5, 10].

On the one hand, our study features a model decomposing responses to combined drought-heat stress relative to single stress responses. While previous research also considered molecular responses to the combination of drought and heat [2, 5, 11] or heat combined with salt, osmotic, cold, light or pathogen stress scenarios [2, 7, 8], we systematically dissect both transcriptomic and metabolomic variables according to their combinatorial response patterns across stress situations and we explicitly show additive vs. interactive effects. Rasmussen et al. [7] pursued a similar goal for transcriptomic data by assigning variables to a set of predefined patterns, whereas our approach assesses additivity by linear modeling. Moreover, since drought and heat are key climatic stress factors affecting plant water relations and have partly opposite regulatory effects on the expression of aquaporin water channels located at the plasma membrane [12, 13], we include loss-of-function mutants of multiple major aquaporins into the analysis in addition to wild-type plants.

On the other hand, we integrate metabolomic and transcriptomic measurements at the data level to explore transcriptome-metabolome relationships across environmental conditions. While previous analyses in the context of abiotic stress combinations focused on sample clustering and condition comparisons [2, 5], data-driven integrated analysis of heterogeneous measurement types has been recognized as a valuable tool in plant sciences and is widely applied [14, 15]. Network-based representations and analysis methods are highly instructive in such systems biology studies [16]. For instance, Hannah et al. [17] looked at correlations between genes and metabolites to identify metabolite mediators of gene regulation; they used different growth conditions including abiotic stresses but no stress combinations. Module or community analysis is a popular approach for exploring gene co-expression networks [18, 19] and is also applicable to correlation networks of heterogeneous entities [20]. The used module finding methods range from network partitioning via hierarchical clustering or label propagation algorithms to the detection of fully connected subgraphs (cliques) [19, 21, 22]. Since correlation networks from omics measurements contain an overwhelming number of variables, an overview of the overall correlation between two measurement types is helpful in identifying experimental factors that drive co-variation as well as the main molecular players that are involved. This is provided by canonical correlation analysis (CCA), which detects the major directions of inter-dataset correlation in an unsupervised and symmetric manner using linear combinations of the original variables, similar as principal component analysis (PCA) [23]. CCA approaches have been performed for various species to investigate transcriptome-metabolome relationships [24, 25] and other data relations [26]. Our study evaluates integrative data exploration both by CCA and by correlation network connections, communities and cliques as a first hypothesis generation step towards discovery of new pathways and functional annotation of uncharacterized metabolic compounds.

## Results

### Different environmental conditions have characteristic expression profiles, which are independent of the presence of major aquaporins

#### Design of drought and heat stress experiments

To analyze molecular responses to abiotic stresses affecting water relations, we collected leaf samples of four-week-old *Arabidopsis thaliana* plants representing six environmental conditions and three genotypes. Environmental conditions were designed to investigate temperature and relative air humidity effects in heat stress as well as combined effects of drought and heat stress. Beside the well-watered control condition (22 °C), we included two variants of heat stress with and without supplemented air humidity (6 h, 33 °C), drought stress (one week without watering), and the combinations of drought stress with each of the two heat stress variants (one week of drought followed by 6 h of heat stress). The samples of a condition were obtained from up to three independent experiments with up to five biological replicates from each genotype (Methods). The genotypes were selected to assess the role of aquaporin water channels in control and stress scenarios and comprised wild-type plants (Col-0) as well as two loss-of-function mutants of major aquaporins from the plasma membrane intrinsic protein (PIP) subfamily, a *pip2;1 pip2;2* double mutant and a *pip2;1 pip2;2 pip2;4* triple mutant (Methods); PIP2;1 (AT3G53420) and PIP2;2 (AT2G37170) are highly abundant aquaporins in both roots and leaves, whereas PIP2;4 (AT5G60660) is a highly expressed root-specific isoform.

#### Analysis of transcriptomic variation

**Fig. 1:**
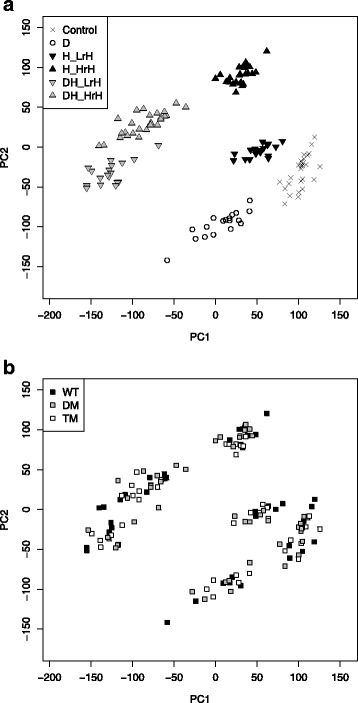
Principle component (PC) analysis-based visualization of transcriptomic data. Projected on the top two PCs (covering 27% and 15% of total variation, respectively), samples are well separated according to environmental condition (**a**) but not according to genotype (**b**). The considered environmental conditions are control, drought (D), heat with low relative air humidity (H_LrH), heat with high relative air humidity (H_HrH), drought stress combined with low air humidity heat stress (DH_LrH) and drought stress combined with high air humidity heat stress (DH_HrH). The considered genotypes are Col-0 wild type (WT), *pip2;1 pip2;2* double mutant (DM) and *pip2;1 pip2;2 pip2;4* triple mutant (TM). PIP2;1 and PIP2;2 are the major aquaporins in leaf tissue

For transcriptomic analysis, microarray measurements were performed (Methods) and give an overview of regulations of 24,603 genes under the different environmental scenarios. The principal component analysis (PCA) visualization of all 135 microarray samples forms clearly separated clusters according to the six environmental conditions; the first two principal components explain more than 40% of the variation (Fig. 1a; Additional file 1: Figure S1). In contrast, genotypes do not show any divergent characteristics (Fig. 1b). Regarding differentially expressed genes between mutants and wild type, the comparatively few changes under the control condition show only a small overlap between *pip* double mutant and *pip* triple mutant (Additional file 1: Figure S2); GO enrichment analysis does not point to any significantly changed biological processes. Differential expression was determined by applying the limma R package [27, 28] on log-transformed data (Methods). Throughout the manuscript, the terms up- and down-regulation imply an absolute log_2_ fold change greater than 1 and an FDR-adjusted *p*-value (p.adj; FDR, false discovery rate) smaller than 0.05.

#### Transcriptional profiles of aquaporin mutants

Notably, no other gene from the major intrinsic protein family including *PIP*, tonoplast intrinsic protein (*TIP*), NOD26-like major intrinsic protein (*NIP*) or small and basic intrinsic protein (*SIP*) genes shows a compensatory effect for the loss of *PIP2;1* and *PIP2;2* at the transcriptional level, neither for the control condition nor for the stress conditions (Additional file 1: Figure S3). This could indicate that compensation for the loss of aquaporins mainly happens at other levels (e.g., translational or posttranslational levels), in specific tissues blurred in the whole leaf extract, in expressed genes missed by the microarray analysis or through redundant water pathways provided by the plant. Interestingly, our analysis reveals that the transcriptomic response profiles to water-related stresses are not affected by the knockout of the aquaporins, although the impact of PIP2;1 or PIP2;2 on water relocation or on developmental processes has been demonstrated [29–31]. Therefore, the mutant samples are included in all condition comparisons without explicit distinction of genotypes. In that way, the (genotype-independent) stress response analysis is strengthened enormously, making the results reliable for follow-up studies due to the large sample basis.

### Plants under combined drought and heat stress maintain major single stress responses but exhibit significant interaction effects

#### Focus of stress combination analysis

To investigate relationships between different abiotic stress conditions, we first decompose the response to a combined drought-heat scenario relative to single stress responses. In particular, we report on interaction effects between drought and heat, which indicate specific regulations in combined stress that deviate from the sum of single stress effects. An interaction with respect to a specific variable means that applying heat stress to well-watered plants does not produce the same change of the variable as applying heat stress to drought-treated plants. While similar investigations have previously been done for phenotypic traits of stressed plants [32], our analysis focuses on molecular responses. In addition to the transcriptomic data from the previous section, we used metabolomic measurements by Fourier transform ion cyclotron resonance mass spectrometry (FT-ICR-MS) on 103 leaf samples; for statistical modeling, we took the 663 masses confirmed by isotope peaks and multiple spectra (Methods). All given *m/z* values refer to the [M-H] ion of negative mode FT-ICR-MS measurements.

#### Differential stress response patterns of transcriptomic and metabolomic variables

**Fig. 2:**
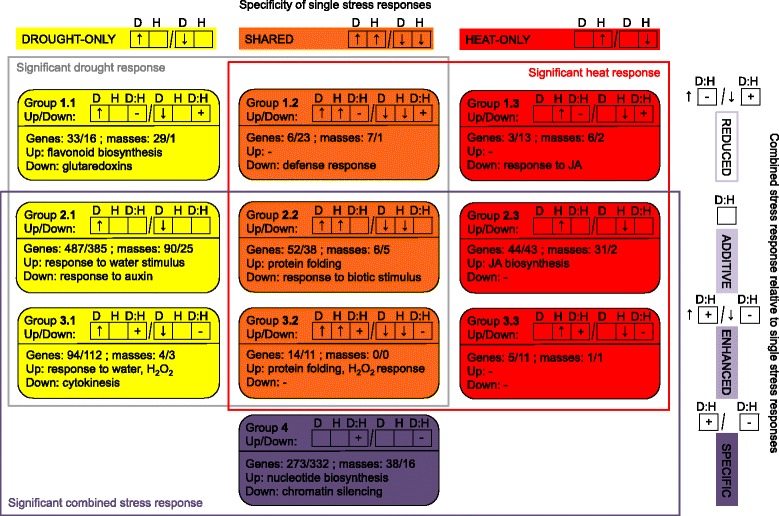
Decomposition of combined drought-heat stress response relative to single stress responses. The ten major response groups of genes and metabolic masses found by linear modeling of transcriptomic and metabolomic data are arranged as a table according to the specificity of single stress responses (columns) and the interaction of single stress responses in combined stress (rows). Each response group consists of two subgroups: the up-regulation part and the down-regulation part. For each subgroup, an arrow code indicates the direction of regulation in the single stresses (D denotes drought, H denotes heat), and a + or - sign denotes a significant positive or negative interaction term (D:H), respectively, which describes the difference between the observed response to combined stress and the combined additive response to single stresses. In the “additive” response groups, there is no significant interaction term, in the “reduced” response groups, the interaction term counteracts the single stress regulation, and in the “enhanced:” response groups, the interaction term enhances the single stress regulation; the specific response group has no single stress regulation but significant interaction. Opposite regulation between drought and heat occurs only in rare cases and is discussed in the main text. For each group, the numbers of up- and down-regulated genes and masses as well as the top significantly enriched processes summarizing the abiotic stress response functions of up- and down-regulated genes are given. For full lists of genes and enriched GO terms and for classification of each variable, see Additional file 2

Categorizing each gene or mass variable according to two criteria, we obtain ten major groups of variables (Fig. 2; Additional file 2). One criterion addresses the specificity of single stress regulation, distinguishing between drought-only, heat-only and shared regulation. The other criterion considers the presence and sign of an interaction effect to describe combined stress response relative to the single stress responses: “additive” response to combined stress corresponds to the combination of drought response and heat response, “enhanced” response indicates a significantly stronger response in combined stress than the additive response, and “reduced” response indicates a significantly weaker response; “specific” response means no significant regulation in single stresses but interaction response in combined stress. Heat conditions with supplemented air humidity are not included in these results; the air humidity aspect will be addressed in the following section.

#### Overall group membership statistics

According to our data, the majority of single stress responses are maintained during combined stress. For drought-only single stress response (groups marked in yellow, Fig. 2), 4% of the genes and 20% of the masses have reduced relevance in combined stress (group 1.1); for heat-only single stress response (groups marked in red), 13% of the genes and 19% of the masses show reduced response in combined stress (group 1.3). Among the 1901 genes having a prominent role in combined stress response (groups bounded by purple line), 55% are additively maintained from single stresses (groups 2.1, 2.2, 2.3), 13% are enhanced (groups 3.1, 3.2, 3.3) and 32% are specific (group 4). Likewise, the percentages for the 222 masses are 72%, 4% and 24%, respectively. Among the single stress response variables that maintain a role in combined stress (additively maintained or enhanced), drought-only players make up a much larger portion (83% of genes, 73% of masses; groups 2.1, 3.1) than heat-only players (8% of genes, 21% of masses; groups 2.3, 3.3), indicating a dominance of the drought contribution.

#### Drought-specific response without heat interaction

The distinct regulation patterns across stresses of the groups in Fig. 2 go along with particular cellular processes, as revealed by GO term enrichment among the up- or down-regulated genes and the functional context of putative metabolic compounds. For the “additive response” groups (groups 2.1, 2.2, 2.3) stress combination does not induce additional regulation. In the drought-only group (group 2.1), up-regulated genes are significantly enriched for many GO terms with previously established connections to drought stress, e.g., response to water deprivation, response to abscisic acid (ABA) stimulus and response to osmotic stress. Five out of the nine clade A protein phosphatases type 2C (PP2Cs), core components of ABA signaling, are in this gene group, two other PP2Cs are shared with heat stress (group 2.2), indicating a partial water deprivation response in heat stress (see discussion of group 2.2 below and next section), and the remaining two members are lowly expressed in leaves (Additional file 1: Table S1); for one of them, ABA-hypersensitive germination 1 (AHG1; AT5G51760), previous work suggests insensitivity to ABA [33]. The down-regulated genes are enriched in processes related to cell division, cell growth, response to auxin and auxin biosynthesis. The 90 up-regulated masses in the group 2.1 include the mass annotated as sucrose (negative mode [M-H] *m/z* 341.1089, C_12_H_22_O_11_) and sorbitol or isomeric sugar alcohols (*m/z* 181.0718, C_6_H_14_O_6_) [34], which may act as osmoprotectants in response to water deficit [9]. Rizhsky et al. [5] also observed up-regulation of sucrose in drought stress and combined drought-heat stress, and Prasch and Sonnewald [2] showed up-regulation in drought stress and in the more extreme heat stress (see next section) with an additive behavior in combined stress. Furthermore, the up-regulated masses putatively include coniferyl alcohol (*m/z* 179.0714, C_10_H_12_O_3_) and its feruloyl malate coupling product (*m/z* 505.1351, C_24_H_26_O_12_). The accumulation of these lignin biosynthesis-related metabolites is consistent with the transcriptional up-regulation of cinnamyl alcohol dehydrogenase (CAD) genes *CAD5* and *CAD6* (AT4G34230 and AT4G37970) as well as the gene encoding cinnamoyl CoA reductase CCR2 (AT1G80820*)* and contrasts with the down-regulation of other cell wall-related genes (Additional file 2).

#### Shared drought and heat effects without interaction

In the additive response group showing the same regulation both in drought and in heat stress (group 2.2), the up-regulated genes are enriched in responses to light intensity, reactive oxygen species (ROS), temperature stimulus and water deprivation. Response to temperature also plays a role in drought stress because stomata closure reduces cooling by transpiration, resulting in elevated leaf temperatures [35]. Response to water deprivation also plays a role in heat stress because heat goes along with enhanced transpiration. Enriched functions down-regulated in both stresses are primarily related to biotic stress response, which is in accordance with previous findings on opposite regulations of abiotic and biotic stress responses [2, 36]. Among the five masses down-regulated in response to both heat and drought, *m/z* 367.3582 (C_24_H_48_O_2_) is annotated as a putative fatty acid: tetracosanoic acid (24:0), a precursor of cuticular waxes, suberin, sphingolipids and phospholipids [37, 38]. A putative increase in wax production is consistent with the cumulative increase of the transcript encoding the Myb domain protein MYB96 (AT5G62470) by drought and heat (Additional file 3); the transcription factor induces cuticular wax biosynthesis, which has been related to drought stress [39, 40]. Three genes of the “additive response” class show contrary regulation between drought and heat (Additional file 2, not shown in Fig. 2). Two of them are up-regulated under drought and down-regulated under heat; they are known as cold- and drought-regulated genes, *LOW TEMPERATURE-INDUCED 30* (*LTI30*; AT3G50970) and *RESPONSIVE TO DESICCATION 29A* (*RD29A*; AT5G52310) [41]. Among the five masses exhibiting contrary regulation between drought and heat, *m/z* 213.1860 (C_13_H_26_O_2_) is annotated as a putative tridecanoic acid (13:0) [34]; our data indicate down-regulation in drought and up-regulation in heat.

#### Enhanced combined stress response

Groups of genes and masses responding to combined stress in a non-additive manner are of special interest since they point to an adaptation of the stress responses that is specific for the combined scenario. In essence, it means that there is a significant contrast in heat response between control plants and plants pretreated with drought. Our model explicitly elucidates which stress response variables undergo such modulation by a simultaneous other stress. The “enhanced response” groups represent synergistic effects of drought and heat, potentially reflecting a highly elevated stress level in the combined scenario that goes beyond the combined effect of single stresses. For example, the drought-induced response to ROS and oxidative stress concentrates in the “enhanced response” category (group 3.1) and not in the “additive response” category (group 2.1), indicating strongly enhanced oxidative stress by additional heat stress, whereas there is no comparable effect in single heat stress. Furthermore, protein folding-related functions are enriched among genes induced by both single stresses and significantly further induced in combined stress (group 3.2). While protein folding is also enriched among genes induced by both single stresses with additive accumulation effect in combined stress (group 2.2), the analysis suggests that many players require particular regulation in the combined stress setting. This could relate to a strong temperature increase by drought-induced stomatal closure in combination with heat treatment.

#### Specific combined stress response

In the “specific response” group (group 4), regulation happens only in combined stress by a drought-heat interaction effect; this excludes accumulation of single stress effects, which dilutes combination-specific genes in stress-versus-control comparisons. Among the up-regulated genes, ribonucleotide biosynthesis-related functional categories are enriched. Among the down-regulated genes, cell division and chromatin silencing functions are enriched. Thus, additional leaf growth-related players are down-regulated as compared to the less severe single drought stress (group 2.1). Consistent with the transcriptional down-regulation of cell division, the induced masses in group 4 assigned to putative plant compounds include the glucoside of methyl cucurbate (*m/z* 387.2024, C_19_H_32_O_8_), a growth inhibitor [42], and tryptophan (*m/z* 203.0826, C_11_H_12_N_2_O_2_), a precursor of the growth hormone auxin [43, 44]. Accordingly, expression of the gene encoding the tryptophan-converting cytochrome P450 79B2 (CYP79B2; AT4G39950) is stronger down-regulated in combined stress than in drought stress (Additional file 3). The up-regulated masses further include two putative glucose-related metabolites, anhydroglucose (*m/z* 161.0455, C_6_H_10_O_5_) and acetyl-glucose (*m/z* 221.0667, C_8_H_14_O_7_), which might relate to the reduced accumulation of putative glucose (*m/z* 179.0561, C_6_H_12_O_6_) in combined stress as compared with drought stress, being part of the “reduced response” group 1.1 described in the following paragraph.

#### Reduced combined stress response

The “reduced response” class contains molecular players where the single stress effects are weakened or completely lost under combined stress. For the drought-only response group (group 1.1), the up-regulated genes are enriched for flavonoid and, in particular, for anthocyanin biosynthesis. Interestingly, four out of the six non-additive genes that have opposite regulation in the single stresses (up-regulated in drought, down-regulated in heat, negative interaction term; Additional file 2, omitted from Fig. 2) also play a role in flavonoid biosynthesis, among them three genes encoding the key enzymes involved in the formation of the flavonoid backbone: chalcone synthase (CHS; AT5G13930), chalcone isomerase (CHI; AT3G55120) and flavanone 3-hydroxylase (F3H; AT3G51240). The expression regulation pattern of these genes nicely integrates the gene regulation patterns of two separate downstream pathways, anthocyanin and flavonol biosynthesis (Additional file 1: Figure S4). Our transcriptomic observations confirm previous findings that anthocyanins accumulate under drought stress but not at high temperatures [45–47]. In the negative ionization mode MS measurements with the used *m/z* range (Methods), anthocyanins were not detected, only two putative flavones (*m/z* 371.1136, C_20_H_20_O_7_, and *m/z* 487.1246, C_24_H_24_O_11_) could be identified in group 1.1. In agreement with our observations, expression of the anthocyanin-related transcription factor *PAP1/MYB75* (production of anthocyanin pigment 1; AT1G56650) was repressed by additional heat compared with sole drought in the results reported by Rizhsky et al. [5] (applying a similar drought/heat regime like in our study). The shared response between drought and heat (group 1.2) contains mostly down-regulated genes, which are enriched for defense response functions, in particular response to the hormones salicylic acid (SA) and jasmonic acid (JA). Relative to the control, the regulation in combined stress is similar to each single stress, suggesting that the default response to the additional stress is suppressed, perhaps to keep immune response at a sufficient level. Consistent with the down-regulation of defense response, a negative regulator of plant immunity, *LIPID TRANSFER PROTEIN 3* (*LTP3*; AT5G59320), is among the genes up-regulated in single stresses; LTP3 also has been shown to enhance ABA biosynthesis [48]. The masses up-regulated in single stresses include the putative raffinose (*m/z* 503.1618, C_18_H_32_O_16_), enabling osmotic adjustment, and the putative aliphatic glucosinolate 6-MSOH (6-methylsulphinylhexyl glucosinolate; *m/z* 464.0725, C_14_H_27_NO_10_S_3_). Previously, a general increase of aliphatic glucosinolates was observed in salinity stress, although 6-MSOH was not detected; aliphatic glucosinolates play a role in plant-herbivore interactions and are correlated with plant water relations [49].

### Dry air is an important component of heat stress and combined drought-heat stress and the primary trigger of metabolomic response

#### The role of air humidity in heat stress

**Fig. 3:**
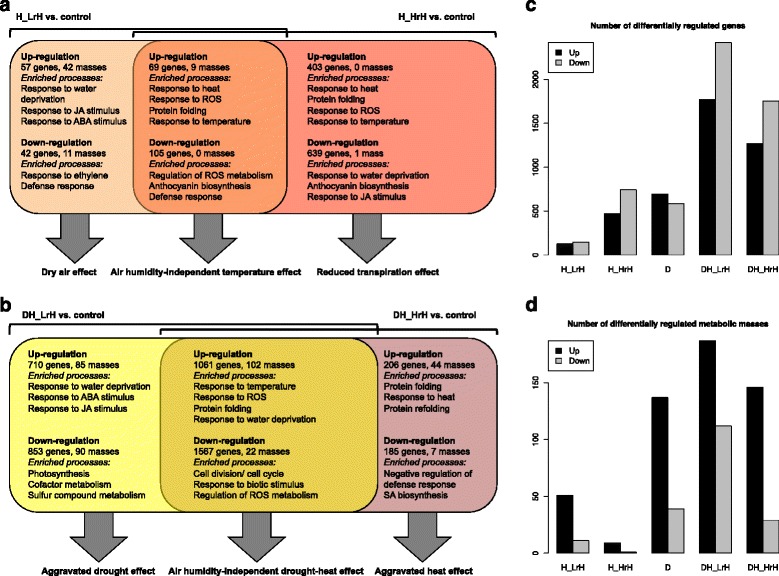
Effects related to ambient air humidity in heat stress and combined drought-heat stress. Venn diagrams of up- and down-regulated variables between low (LrH) and high air humidity (HrH) settings and major enriched GO terms in each group are shown for heat stress (H; **a**) and combined stress (DH; **b**). In addition, the total number of differentially regulated variables under each stress condition relative to the control condition is depicted for transcriptomic (**c**) and metabolomic data (**d**). The relatively smaller number of down-regulations as compared with up-regulations in the metabolic data is related to the data acquisition: low abundances cannot be detected and are therefore not accurately quantified but set close to the detection threshold

A heat episode enhances the VPD of the ambient air provoking a water deficit (“low air humidity” setting: 33 °C/ 37% relative humidity, 3.17 kPa VPD). To better understand the components of heat stress responses and their modulation by precedent drought stress, air humidity was supplemented in a parallel set of heat experiments to keep the VPD constant and thereby avoiding a parallel water deficit in the ambient air (“high air humidity” setting: 33 °C/ 84% relative humidity, 0.79 kPa VPD). Thus, the components of heat stress can be dissected in a systematic way into a temperature-related and into a humidity-dependent part by comparing the molecular responses to heat between high and low air humidity settings (Fig. 3a; Additional file 4). In addition to changes in the ambient air, heat will also affect soil evaporation. However, in contrast to the strong effects on VPD, the enhanced temperature led to small differences in evaporation of water from soil during the short 6 h heat episode with different air humidity settings (Additional file 1: Figure S5a). With regard to leaf water content, samples of control plants were close to the heat-treated specimen except for the rosette leaves after the drought-heat episode with low air humidity (DH_LrH), which showed a reduction by about 10% (Additional file 1: Figure S5b).

#### Transcriptional up-regulation related to low air humidity

The first group consists of genes that are up-regulated in the low air humidity heat stress but not in the high air humidity heat stress. This group represents a component of heat stress that cannot be attributed to ambient temperature (which was the same in both settings) but is due to the decreased relative air humidity during heat (“dry air” effect). Increased transpiration by dry air is expected to induce a water deficit in leaves, compared with the high air humidity setting (Additional file 1: Figure S5b). Indeed, the genes in this group are significantly enriched in functions related to drought, namely hyperosmotic salinity response (p.adj = 0.00289) and response to water deprivation (p.adj = 0.00474). The down-regulation of the same stress responses after eliminating the dry air effect (i.e., in the high air humidity-only group) further supports this relationship. JA-related processes are transcriptionally up-regulated in the dry air-specific group and down-regulated at supplemented air humidity (Additional file 4). While JA biosynthesis and response have previously been associated with heat stress response and thermotolerance in *Arabidopsis* [50–52], our data indicate that this relates to the dry air component under heat stress.

#### Transcriptional up-regulation related to temperature

The overlap between high and low air humidity heat stress responses can be considered as purely temperature-related effect for the ambient condition of 33 °C. The top enriched processes among the up-regulated genes are response to heat (p.adj = 3.36e-29), responses to hydrogen peroxide and ROS (p.adj = 3.72e-24 and p.adj = 1.23e-20), and protein folding (p.adj = 5.58e-19). The genes mainly include heat-induced transcription factors such as *MULTIPROTEIN BRIDGING FACTOR 1C* (*MBF1C*; AT3G24500) and heat shock factor (*HSF*) genes of the A and the B subfamilies. Further members are genes encoding heat shock proteins (HSPs) and other protein chaperones. According to previous studies, these are major players in response to heat stress; *MBF1C* is required for elevated expression of *HSF*s of the B family, salicylic acid- and ethylene-responsive genes as well as the trehalose pathway, and *HSF*s of the A family are linked to *HSP*s and ascorbate peroxidase-related heat stress responses [53–55]. HSPs prevent protein misfolding and aggregation [56, 57]. In line with that, our analysis suggests that induced expression of *MBF1C*, *HSF*s and genes encoding protein chaperones is mainly dependent on the high temperature component of heat stress.

#### Transcriptional up-regulation related to high air humidity

The remaining third group represents a response that is specific to heat with high relative air humidity. The induced genes include additional *HSF*s and *HSP*s, which were also detected in previous heat stress experiments using higher temperatures (Additional file 1: Table S2). A plausible reason for the more severe temperature response at maintained VPD is an elevation of the leaf temperature caused by lower transpiration relative to the dry air-inducing heat application. The molecular response highlights that air humidity is a relevant factor influencing the temperature response in addition to the ambient temperature. Our observation agrees with the different extent of heat shock responses under mild and severe heat stress reported by Prasch and Sonnewald [2] and the accumulation of ROS and heat shock proteins reported by Yang et al. [58]. Remarkably, all responses to high relative air humidity are temperature-related.

#### Transcriptional down-regulation related to heat stress components

All three groups show down-regulation of biotic stress defense genes, confirming the increased pathogen susceptibility often observed under abiotic stress [36]. The down-regulated genes both in the second and third group are enriched for biosynthetic processes of anthocyanin-containing compounds (p.adj = 9.76e-8), in accordance with the lack of a drought response (which would induce anthocyanin biosynthesis, see above).

#### Air humidity-related transcriptional up-regulation in combined drought-heat scenarios

To check whether preceding drought stress modulates the air humidity effects under heat, an equivalent comparative analysis of air humidity effects is shown for the combined drought-heat stress (Fig. 3b, Additional file 4). Compared with the heat stress scenario, the gene expression response we observed in the drought-heat combination is larger for the low air humidity-specific part and smaller for the high air humidity-specific part. The genes up-regulated in both air humidity settings are enriched in central drought categories including response to water deprivation and response to ABA stimulus as well as the central high temperature categories that already occurred in the heat stress comparison (Fig. 3a). For the up-regulated genes unique to the low air humidity setting, biological processes related to water deprivation, ABA stimulus and JA response are leading, which is similar to the dry air effect in heat stress response. However, more than six times as many key regulators are detected, including the PP2C gene *E GROWTH-REGULATING 1* (*EGR1*; AT3G05640), *SUCROSE NONFERMENTING1-RELATED PROTEIN KINASE 2.6/ OPEN STOMATA 1* (*SnRK2.6*/ *OST1*; AT4G33950), *ABA-RESPONSIVE ELEMENT BINDING FACTOR 4* (*ABF4*; AT3G19290) and other genes encoding ABA-induced transcription factors, the JA biosynthesis gene *ACYL-CoA OXIDASE 1* (*ACX1*; AT4G16760) as well as the JA response genes encoding the jasmonate-zim-domain proteins JAZ1 and JAZ4 (AT1G19180 and AT1G48500) and JA-ASSOCIATED MYC2-LIKE 1 (JAM1; AT2G46510). The transcriptional pattern of *JAZ1* and *SnRK2;6/OST1* was independently verified by RT-qPCR to validate the microarray data (Additional file 1: Figure S6). These results suggest that preceding drought stress aggravates the dry air-induced drought effect with respect to ABA- and JA-related biological processes under heat stress. The up-regulated genes unique to the high air humidity setting are enriched with respect to protein folding and response to heat, which is consistent with the heat stress analysis, but the unique response is less pronounced than in heat stress, indicating an interaction with the drought stress response. Indeed, approximately 42% of the high air humidity-specific up-regulated genes in heat stress are not at all up-regulated in combined stress with high air humidity; thus, preceding drought stress has partly counteracting effects on the heat response. GO enrichment analysis of these genes yielded significant processes related to heat response, cell wall, hexose stimulus response, extracellular region and water channel activity. The water channel genes that are transcriptionally up-regulated in single heat stress but not in combined stress include *TIP1;1*, *TIP2;1*, *PIP1;5* and *PIP2;7*. During drought, these genes are down-regulated, supposedly to reduce water flow through membranes and maintain leaf turgor [12]; apparently, this effect dominates in the combined stress scenario.

#### Air humidity-related transcriptional down-regulation in combined drought-heat scenarios

The down-regulated genes shared between both humidity settings are strongly enriched in cell cycle functions, probably reflecting reduced growth due to drought. The genes specifically down-regulated in low air humidity are dominated by photosynthesis-related functions, which confirms that drought stress hampers photosynthesis through stomatal closure and dehydration of mesophyll cells. The fact that transcriptional down-regulation of photosynthesis genes manifests itself under combined stress but is not yet visible under drought stress at control temperature (Additional file 2) confirms the observation by Zhao et al. [59] that high temperature sooner leads to a negative carbon balance in trees under drought; our analysis suggests that this is particularly relevant for heat with low relative air humidity. In summary, the comparison of the different air humidity settings in heat and combined stresses illustrates the temperature and dry air effects associated with heat stress.

#### Comparison of transcriptomic and metabolomic air humidity effects in abiotic stress responses

Interesting parallelisms and differences between transcriptomics and metabolomics regarding the air humidity effects under different stress conditions are revealed considering the overall extent of stress responses, quantified by the number of differentially regulated genes or masses relative to the control condition (Fig. 3c and d). In both data types, without supplementary air humidity, drought stress induces a larger response than heat stress and combined stress induces a larger response than single stresses. With increased air humidity, the extent of the transcriptomic response is increased in heat stress but reduced in combined drought-heat stress (Fig. 3c). The latter could reflect an alleviated drought effect by reduced transpiration; the smaller overlap between drought stress and high air humidity combined stress relative to the overlap between drought stress and low air humidity combined stress additionally confirms this assumption (Additional file 1: Figure S7) [60]. Furthermore, as indicated above, the heat effect is partially counteracted by preceding drought. However, the combined stress response under high air humidity still exceeds the single stress responses. The metabolomic response is reduced by increased air humidity both in combined stress and in single heat stress (Fig. 3d). While the former agrees well with the finding from transcriptomic data, the latter is opposite to the transcriptomic data. These observations stay the same when varying the preprocessing or fold change thresholds for the metabolomic data. In fact, no mass is specifically up-regulated and only one mass is specifically down-regulated in heat with high air humidity, in contrast to a large number of low air humidity-specific regulations (Fig. 3a). The results suggest that the dry air effect by far exceeds the temperature effect in metabolomic response to heat stress.

### Correlated changes between transcriptomics and metabolomics reveal putative players in abiotic stress responses

#### Transcriptome-metabolome data integration

Both transcriptomic and metabolomic data have shown significant responses to stress scenarios, revealing the involvement of known, annotated metabolic pathways and biological processes. In contrast to the previous sections, we now aim at investigating relationships between the transcriptome and the metabolome in a data-driven manner without relying on prior knowledge of cellular processes and metabolite-gene connections. For that purpose, we perform data level integration of transcriptomic and metabolomic measurements across all environmental conditions, using those 56 samples where both measurement types are available. We explore major intrinsic variability components shared between the transcriptome and the metabolome as well as fine-grained co-regulatory relationships between gene expression and metabolite abundance.

#### Shared variation between transcriptome and metabolome

**Fig. 4:**
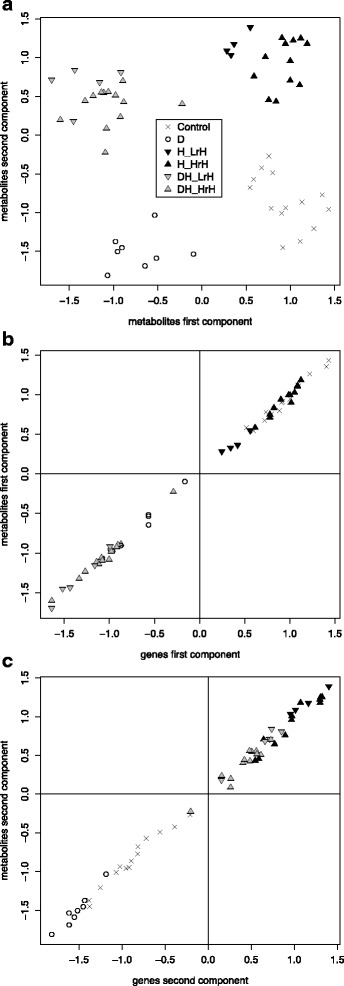
Canonical correlation analysis (CCA) integrating transcriptomic and metabolomic data. Projection of metabolomic samples onto the first two CCA components shows a clear separation of drought, heat, drought-heat and control conditions (**a**). The first CCA component shows a high correlation between transcriptomic and metabolomic projections (Pearson coefficient 0.990) and separates drought samples from non-drought samples (**b**). The second CCA component also shows a high correlation between transcriptomic and metabolomic projections (Pearson coefficient 0.959) and separates heat samples from non-heat samples (**c**)

A well-known approach to find common variation between two data types is CCA, a dimension reduction method that generalizes PCA to two data sources with two different feature spaces. It finds projection directions of the two feature spaces such that the projected data show maximal correlation between the two data sources. To be able to handle feature spaces with more variables than the number of paired samples, we used the regularized CCA method implemented in the mixOmics R package [23, 61]. Similar to PCA, the result is a sequence of components in the order of decreasing correlation. Each component consists of a pair of canonical variates, one for each feature space. Each canonical variate is a linear combination of variables in the original feature space, and it is uncorrelated to canonical variates earlier in the sequence. Fig. 4a shows the projection of the metabolomics data onto the first two components obtained from regularized CCA for the top 100 variables with largest variance from each data type. Interestingly, the first component separates drought from non-drought samples and the second component separates heat from non-heat samples, with high transcriptome-metabolome correlations (Fig. 4b, c). Since the condition labeling was not available to the method, this result indicates in an unbiased way that transcriptome and metabolome mainly correlate with respect to the drought and heat response; in contrast, air humidity response differs between data types (see also previous section).

#### Main drought response players relating transcriptome and metabolome

To interpret the CCA components regarding potential functional connections between genes and metabolic compounds, we consider correlations between the canonical variates and each original variable of the two data types. For the first component (Fig. 4b), the top metabolic mass positively associated with drought is the putative sucrose (*m/z* 341.1089; correlation with first component: −0.972, p.adj = 6.05e-33). The top gene encodes the cell wall/vacuolar inhibitor of fructosidase C/VIF1 (AT1G47960), a putative inhibitor of cell wall and vacuolar invertases [62] (correlation −0.981; p.adj = 2.38e-38). Another top gene encodes the cell wall invertase *CWINV5* (AT3G13784; correlation −0.968, p.adj = 1.01e-32). The enzyme catalyzes the conversion of sucrose into glucose and fructose and is predicted to be located in the cell wall [63]. Overexpression of a cell wall invertase gene from *Chenopodium rubrum* in tomato has been shown to increase water use efficiency and drought tolerance [64]. Prasch and Sonnewald [2] observed up-regulation of sucrose and *CWINV5* in combined drought and heat stress; in mild drought stress, only sucrose was up-regulated. Sucrose or *CWINV5* themselves are sufficient to separate drought from non-drought samples in our data (Additional file 1: Figure S8, left panel).

#### Sucrose inversion networks

**Fig. 5:**
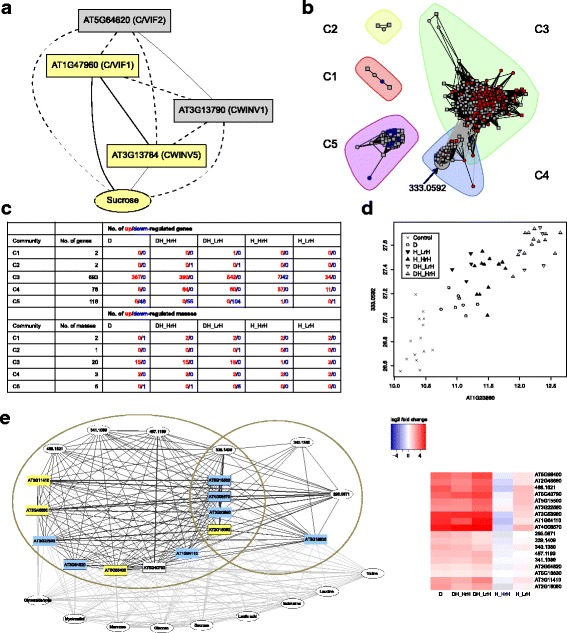
Correlation networks between genes (boxes) and metabolomic features from FT-ICR-MS data (ellipses). Positive correlations >0.75 (solid lines) form two groups of sucrose inversion-related entities (marked in *yellow and gray*, respectively); members from different groups are negatively correlated (dashed lines; **a**). The global network of topmost positive correlations (>0.85) between genes and metabolomic masses from the non-targeted metabolomics measurements consists of five communities (**b**). *Red and blue colors* indicate up- and down-regulation under drought stress, primarily occurring in the communities C3 and C5, respectively. The marked metabolomic mass, a putative glycerophosphoinositol, is the most connected mass within community C4, which in contrast to other communities mainly represents heat-related response (**c**). The mass and its top correlated gene together separate different environmental conditions, with gradually more induction from control to drought, heat and combined stress (**d**). A subnetwork of closely interconnected genes and masses (*black edges*; the ellipses mark fully connected groups) suggests drought-related functions of unassigned masses and unknown genes (**e**). Known genes related to response to water deprivation are annotated by *yellow color*, known genes having functions as transport and membrane proteins are annotated by *blue color*. *Gray edges* indicate correlations >0.85 to metabolites from GC-MS data; only metabolites with at least ten gene connections are shown. The heatmap shows that stress response profiles are quite homogeneous for all subnetwork members

To check how these top variables relate to the three other known cell wall invertases and the other cell wall invertase inhibitor annotated in TAIR, we computed correlations based on our paired dataset. Interestingly, there are two anti-correlated submodules, each of which consists of strongly positively correlated variables (Fig. 5a), whereas the genes *CWINV2* (AT3G52600) and *CWINV4* (AT2G36190) did not show absolute correlations stronger than 0.75 to any of the other variables. Remarkably, this structure of sucrose-gene and gene-gene relationships is exactly reproduced in an independent analysis of targeted sucrose content data (from validation measurements using gas chromatography mass spectrometry, GC-MS) of drought-treated and control samples and their corresponding transcriptomes, although less than 50% of the samples overlapped with the large FT-ICR-MS-based integration study (Methods, Additional file 5, Availability of data). One submodule consists of sucrose, *CWINV5* and *C/VIF1*. The other submodule comprises *CWINV1* and *C/VIF2*. The co-regulation of *CWINV5* and *C/VIF1* across conditions suggests that *C/VIF1* is a specific inhibitor to *CWINV5*, necessary to fine-tune the activity of the intrinsically stable cell wall invertase proteins [62]. Likewise, *C/VIF2* might be specific to *CWINV1*. An alternative hypothesis in line with the observed correlations could link the anti-correlated invertase/ invertase inhibitor pairs as functional, interacting entities. Eventually, the anti-correlation of such two submodules could indicate complementary roles of invertases in drought stress and control conditions. Consistently with our finding on the transcriptional regulation of *CWINV1* and *CWINV5* per se, Prasch and Sonnewald [2] had reported strong down-regulation of *CWINV1* in conjunction with the above-mentioned up-regulation of sucrose and *CWINV5* in combined stress.

#### Main heat response players relating transcriptome and metabolome

For the second CCA component, associated with heat (Fig. 4c), the top gene is of unknown function (AT2G31560, correlation 0.923), and the second one (AT1G55960, correlation 0.916) encodes a polyketide cyclase/dehydrase and lipid transport superfamily protein putatively involved in the myo-inositol hexakisphosphate biosynthetic process (GO:0010264); phospho-inositide signaling has been linked to heat stress [65, 66]. Projecting the data on the topmost functionally annotated gene of the first and the second component already yields a condition separation that is almost as good as the CCA projection (Additional file 1: Figure S8, right panel; Fig. 4a), suggesting these genes as suitable biomarkers for drought and heat scenarios. The top correlated metabolic mass of the second component is *m/z* 259.0590 (correlation 0.884). The observed data suggest that this metabolic feature and the un-annotated gene AT2G31560 have a so far unknown functional role in heat stress response.

#### Global correlation network for metabolite characterization

To characterize more metabolic masses with respect to putative biological contexts, we investigated a global network of the strongest mass-gene correlations from our data (Fig. 5b). It contains all genes and masses with gene-mass correlations greater than 0.85 (893 genes, 31 masses). The visualization further includes and utilizes all mass-mass and gene-gene correlations with correlation coefficients greater than 0.85 and adjusted *p*-values smaller than 1.0e-5. The community structure of the network was determined by the label propagation algorithm implemented in the igraph R package [67, 68] and yielded five groups, each consisting of densely connected entities. Interestingly, these five network communities reflect characteristic condition-specific regulation patterns (Fig. 5c). The largest community, C3, is dominated by genes and masses up-regulated in response to drought-related stresses, including the dry air effect of heat application, whereas down-regulation of genes dominates in heat stress with high relative air humidity. In contrast, community C4 includes a large fraction of genes that are up-regulated in heat stress with high relative air humidity and other heat-related stresses but rarely in single drought stress. Two masses of the C4 community are linked to the C3 community; they show an up-regulation in response to all stresses. The C5 community shows the opposite pattern to C3, namely down-regulation in drought-related stresses (Fig. 5b). The functional analysis of community genes is in line with the stress response profiles, with response to water deprivation being the top enriched GO category in community C3 (p.adj = 2.74e-24) and photosynthesis light reaction the top category in community C5 (p.adj = 1.72e-6; Additional file 6). Community C4 shows the most significant enrichment in response to radiation (p.adj = 4.11e-4) and mRNA metabolic process (p.adj = 0.0024). Specifically, the C4 community genes that are up-regulated under heat with high air humidity are enriched in RNA splicing and heat response genes (including heat shock factors, transcription factors and enzymes).

#### Investigation of a putative metabolite related to heat stress response

In addition to the putative sucrose, which has the largest number of edges in the correlation network and is part of the C3 community, only one of the masses yielded potential matches to known metabolites from KEGG via the MassTRIX platform [34], namely *m/z* 333.0592 in community C4, which represents heat-related response. Interestingly, *m/z* 333.0592 was assigned to be a putative glycerophosphoinositol (GroPIns; C_9_H_19_O_11_P; KEGG:C01225) or one of three other isomers differing in the arrangement of phosphate, glycerol and sugar group. GroPIns and its derivatives are involved in gene expression and transcription factor activation controlling cell proliferation and inducing cell differentiation in mammals [69–71]. In plants, GroPIns function has not been investigated yet, although myo-inositol and phosphatidyl-inositol signaling has been related to environmental stress [65, 66]. The genes positively correlated with *m/z* 333.0592 are involved in mRNA processing and transcriptional regulation, suggesting that *m/z* 333.0592 might play a role in *Arabidopsis* gene expression resembling GroPIns in mammals. The top positively correlated gene is AT1G23860 (correlation 0.888), which encodes a serine-arginine rich protein involved in nuclear precursor mRNA processing. Remarkably, the correlated changes between *m/z* 333.0592 and AT1G23860 together allow a clear separation of different environmental conditions, with gradually more induction from control to drought, heat and combined stress (Fig. 5d). This suggests that the mass-gene pair could be used to discriminate these abiotic conditions, potentially indicating the extent of transcriptional regulation of specific heat response processes. Both the mass and the gene transcript are present at high levels in cells: the mean level of the mass is among the top 5% of all masses in the FT-ICR-MS dataset, the mean level of the gene transcript is among the top 15% of all genes in the microarray dataset.

#### Dense modules of drought response players

Finally, we investigated dense modules in the network with the aim to determine candidates with potentially stress-relevant functions among the yet uncharacterized masses. To reduce the overload of genes to the most relevant ones, we included only the top three correlated genes for each mass as nodes in the network (Additional file 1: Figure S9). Based on the positive correlation edges (correlation >0.85, p.adj < 1.0e-5), fully connected subnetworks (cliques) that are not contained in other cliques were detected; 27 of these cliques contained metabolic masses. For additional information, we also computed correlations between the genes of a clique and the variables from our targeted metabolomics dataset, building another set of network edges (Additional file 5). One of the cliques consists of four masses from the non-targeted analysis and eleven genes; it overlaps with another clique of three masses and five genes, sharing one mass and four genes (Fig. 5e). Since all genes with available annotation in these overlapping cliques have roles in transport or in response to water deprivation (e.g., as transcriptional regulator, signaling regulator or dehydrin chaperone), we propose that the included masses and the unknown gene AT5G40790 are closely related to drought response processes, which is further supported by the stress response profiles of clique members shown in the heatmap (Fig. 5e). According to ChemSpider [72], the mass overlapping between the two cliques, *m/z* 339.1409, could be a disaccharide from two amino sugars derived from glucose (C_12_H_24_N_2_O_9_). Like the other nodes in the two cliques, this metabolic feature is up-regulated in all stresses having a drought component, including a slight up-regulation in heat stress with dry air; it is down-regulated in heat stress with high air humidity. In agreement with that, the node shares its gene connections with carbohydrate osmolytes from the targeted metabolomics data (glucose, mannose, sucrose, inositol, glyceraldehyde) and the putative sucrose mass from the non-targeted metabolomics dataset *(m/z* 341.1089). This suggests the possibility of osmotic stress response function for that mass. Furthermore, the branched-chain amino acids (leucine, isoleucine and valine) and 3-phenyl lactic acid involved in phenylalanine metabolism are connected to all or all but one gene of the subnetwork (Fig. 5 e). The accumulation of branched-chain amino acids under drought stress has been observed previously [73].

#### Dense module down-regulated under drought stress

Another clique has the opposite stress regulation pattern to the cliques in Fig. 5e: strong down-regulation in drought-related stresses, slight down-regulation under heat with dry air, slight up-regulation or no regulation under heat with high air humidity (Additional file 1: Figure S10). It consists of seven genes and two masses, *m/z* 449.1121 and *m/z * 549.2300; six genes are annotated with functions in the extracellular compartment and in transport; the seventh gene encodes a transmembrane protein of unknown function. Unambiguous annotation of the masses is not possible; the data propose some (potentially indirect) relationship to membrane processes required under heat stress and normal growth conditions but not under drought stress. The most correlated metabolite from the targeted metabolomics data is galactinol, having correlation >0.85 to four out of the seven genes and correlation >0.75 to all seven genes; the top correlation is to AT4G16980, a member of the (extracellular) arabinogalactan-protein family (Additional file 5). Accumulation of galactinol has been described as a heat stress response [74].

The results exemplify how a data-driven joint analysis of transcriptomic and metabolomic profiles substantiates current knowledge and leads to new hypotheses regarding abiotic stress responses in *Arabidopsis* plants.

## Discussion

### Simultaneous heat stress affects flavonoid biosynthesis, oxidative stress and protein folding in response to drought stress but not ABA signaling and osmotic regulation

Decomposition of combined stress responses revealed interactions between drought and heat responses both at transcript and at metabolite level, suggesting that single stress responses are partly adjusted during the combined stress situation. The molecular response to drought stress is characterized by ABA signaling, osmolyte biosynthesis, macromolecule protection, cuticular wax synthesis, reactive oxygen species detoxification and flavonoid accumulation [75, 76]. All these response processes were observed in our experiments, and we additionally investigated whether and how they are changed by simultaneous application of heat stress (Fig. 2; main text). In particular, flavonoid biosynthesis is adversely affected by the stress combination, whereas many oxidative stress response and protein folding genes are specifically induced. In contrast, ABA signaling and osmolyte regulation (including accumulation of sucrose and putative sugar alcohols) in response to drought stress are not altered by addition of heat stress. These findings have implications in stress tolerance breeding. The gene categorization resulting from our analysis gives hints on the suitability of a gene target regarding tolerance in a range of climatic scenarios. For instance, optimization of osmoprotection seems more promising than optimization of flavonoid biosynthesis in areas where drought is often accompanied by heat waves. Likewise, it might be useful to consider the enhanced relevance of oxidative stress response and protein folding under combined stress. A clean distinction between enhanced, additive and reduced response groups is required for such considerations. Therefore, an interaction analysis of stress combination has been employed here, complementing the relations provided by classical Venn diagrams. However, the basic molecular response patterns we observed are consistent with previous stress combination studies, as elaborated in the case of sucrose and different cell wall invertases, for instance [2, 5].

### Combination-specific stress regulatory effect suggests decoupling of ribosome biogenesis and growth

Regarding transcriptomic effects occurring only in the severe condition of combined stress, we observed an up-regulation of ribonucleotide biosynthesis and a down-regulation of cell division processes (Fig. 2, group 4). The simultaneous occurrence of ribonucleotide biosynthesis, including ribosome biogenesis, and cell division inhibition might suggest that plants prepare the translational machinery for the recovery phase where more protein biosynthesis will be needed [77]; a recent study in a maize population also showed a negative correlation between leaf growth and transcript levels of genes encoding for ribosomal proteins, which indicates that the energy-consuming process of ribosome biogenesis is minimized for growth [78]. In agreement with this, a transcriptional down-regulation but unchanged protein abundance for the majority of ribosomal proteins was observed during *Arabidopsis* leaf growth, suggesting improved ribosomal protein stability [79]. This is confirmed by a decrease of ribosome protein degradation rates with increasing growth rates in *Arabidopsis* [80]. Similar to our finding, Noir et al. reported elevation of ribosomal gene expression in conjunction with jasmonate-inhibited cell proliferation and leaf growth in *Arabidopsis* and interpreted it as a stand-by mode of the cell [81]. Thus, our finding corroborates previous results; proteomic, transcriptomic and genomic evidence fit together. Our metabolomic data additionally suggest relationships to metabolic pathways involving growth hormones and growth inhibitors.

### Relative air humidity modulates the relative extent of drought and temperature components in heat stress responses

The response to heat stress is of major interest for climate change scenarios and has been extensively studied at the transcriptome level [2, 3, 52, 54, 56, 57]. The incorporation of varying air humidity as an experimental factor in our study revealed a prominent drought component of heat stress responses in leaves (Fig. 3a and b), explaining in particular JA- and ABA-related responses [52]. This heat-related drought effect also led to a clear dominance of drought responses under combined drought-heat stress when no supplementary air humidity is added (Fig. 2). The majority of gene up- or down-regulations in combined stress relative to the control condition come from cumulative effects of modest drought and heat regulations, reflected by the larger number of genes in comparison to control conditions (Fig. 3c) than in the respective groups of the decomposition model (Fig. 2). Nevertheless, the drought contribution is stronger than the heat contribution: among the genes up-regulated in combined stress relative to the control, 75% are higher expressed in drought than in heat; among the genes down-regulated in combined stress, 77% are lower expressed in drought than in heat (Additional files 3 and 4).

On the other hand, relative air humidity may also have an influence on the temperature component of the heat stress response. We showed that high air humidity has no significant other effect than increased temperature response (Fig. 3a and b). For combined stress, the role of air humidity can be summarized as follows: low air humidity enhances the transcriptomic drought response and high air humidity enhances the transcriptomic heat response. Our data also contribute to explaining discrepant observations in previous transcriptomic studies on drought-heat combinations: while Rizhsky et al. [5] used settings close to our low air humidity condition and found a dominance of drought-related effects in combined stress, Prasch and Sonnewald [2] observed a dominance of heat responses after applying a longer but less extreme heat stress than Rizhsky et al. and maintaining the relative air humidity at 60%, which makes the setting closer to our high air humidity condition. From a physiological perspective, an increase in relative air humidity gradually reduces transpiration (Additional file 1: Figure S11). As a consequence, cooling is less effective, aggravating heat effects, but excessive water loss is prevented, alleviating drought effects. Taken together, the results support the hypothesis that air humidity modulates the prevalence of drought and temperature components of the response to heat stress. The air humidity aspect is inherently connected with heat stress due to the temperature-dependent change of the vapor pressure of water in air. Our analysis shows that molecular responses in the plant are clearly affected by this change, resulting either in enhanced temperature response or in activation of drought response.

### Metabolism is sensitive to drought but remains largely unaffected by temperature stress due to massive transcriptomic measures for protein protection

Comparing the relative responses to low and high air humidity heat stresses between metabolome and transcriptome, the most striking observation is that the metabolome primarily responds to the enhanced drought due to lower air humidity, whereas the transcriptome mainly responds to temperature increase at high air humidity conditions (Fig. 3a; Additional file 1: Figure S12). Such a finding would not be detectable from separate studies, emphasizing the potential and importance of a side-by-side analysis of different omics levels. The results suggest that a temperature heat episode requires massive transcriptional changes but per se has comparatively little effect on metabolite levels. A huge part of the transcriptomic up-regulation concerns proteins that ensure proper protein folding (e.g., chaperones), which – if working well – would not have consequences on metabolic pathways. Thus, a possible biological interpretation is that protein protection as a major temperature response allows for a close-to-normal metabolism. In contrast, the heat-associated increase in VPD (dry air) accounts for the majority of metabolomic heat stress responses. Since dry air might provoke drought in leaves, we expect that the metabolomic response partly resembles that of a drought stress scenario. Indeed, 21% of the masses up-regulated only in heat with low air humidity and not in heat with high air humidity are also up-regulated in drought (Additional Files 2 and 4). Among them, we find *m/z* 353.1031, putatively a lignan (C_20_H_18_O_6_) that is synthesized from coniferyl alcohol; putative coniferyl alcohol is also accumulated in drought (Results). Some drought-induced osmoprotectants like the putative raffinose (*m/z* 503.1618) are shared between the low and high air humidity responses both in heat stress and in combined stress, indicating that high temperature may also induce osmotic responses. In fact, accumulation of raffinose has previously been observed both after heat shock and after cold shock [82], highlighting its general role. In summary, our results support a hypothesis of drought sensitivity and temperature insensitivity of the metabolome. Still, combined stress shows a substantial number of regulated metabolites under high air humidity only (Fig. 3b), among them putative lignans, flavonoids and glucosinolates (Additional file 4). Their role could potentially be related to differences in drought responses between the two air humidity settings.

### Putative glycerophosphoinositol is an mRNA processing-related biomarker of heat stress and may be metabolized via mammal-like pathways

A substantial part of the *Arabidopsis* metabolome is still uncharacterized [83]. By exploring data-driven relationships between metabolomic and transcriptomic features, it is possible to develop hypotheses on the nature of unknown compounds or to suggest stress-related biomarkers (e.g.*,* drought and heat biomarkers: Fig. 4; Additional file 1: Figure S8). Using a global correlation network primarily based on strong gene-mass correlations, we identified *m/z* 333.0592 together with the mRNA processing gene AT1G23860 as a biomarker of abiotic stress, separating drought, heat and combined scenarios (Fig. 5d) and representing in particular heat response (Fig. 5b, c). We employed correlation analysis as a tool to identify candidate genes potentially involved in the metabolism of *m/z* 333.0592. Database search with MassTRIX [34] suggested that *m/z* 333.0592 may be sn-glycero-3-phospho-1-inositol (GroPIns; KEGG:C01225) or an isomeric molecule. In mammals, the degradation of GroPIns is catalyzed by the GroPIns phosphodiesterases (GDEs) GDE1 or GDE3, producing either myo-inositol and glycerol phosphate or myo-inositol phosphate and glycerol. Negatively correlated candidate genes are AT4G34930 (correlation −0.863), encoding a phosphodiesterase of the phospholipase C (PLC)-like superfamily to which mammalian GDE3 also belongs, as well as AT5G63990 (correlation −0.859) and AT4G05090 (correlation −0.847), both encoding inositol monophosphatases that could further convert myo-inositol phosphate to myo-inositol. Among the 12 PLC-like phosphodiesterases annotated in TAIR [4], we find only strong anti-correlations with *m/z* 333.0592, no strong positive correlations. Taken together, a two-step pathway from GroPIns to myo-inositol could exist in *Arabidopsis thaliana*, and its down-regulation under stress could contribute to the accumulation of GroPIns.

Interestingly however, the targeted metabolomics data show that myo-inositol is up-regulated in drought vs. the control condition (Additional file 1: Figure S13). This suggests that other pathways than the GDE3-like pathway lead to an accumulation of myo-inositol under stress. One option would be an equivalent to the mammalian GDE1 pathway, which produces myo-inositol in a single step directly from GroPIns, releasing sn-glycerol-3-phosphate (PLD-like phosphodiesterase activity). A TAIR search [4] yielded 39 phosphodiesterases, among them 13 glycerophosphodiester phosphodiesterases which could catalyze a GDE1-type reaction (EC 3.1.4.44). Two of them, the glycerophosphodiester phosphodiesterases *GDPD4* and *GDPD3* (AT1G71340 and AT5G43300), are significantly positively correlated with *m/z* 333.0592 (0.827 and 0.708, respectively), indicating that a GDE1-like pathway potentially exists in *Arabidopsis* and is induced upon abiotic stresses, in contrast to the putative GDE3-like pathway, where down-regulation is observed (Additional file 1: Figure S14). Another pathway to myo-inositol production is the conversion of glucose-6-phosphate via myo-inositol-3-phosphate (EC 5.5.1.4, 3.1.3.25; KEGG:00562). The myo-inositol-phosphate synthase (MIPS) genes *MIPS1* (AT4G39800), *MIPS2* (AT2G22240), *MIPS3* (AT5G10170) catalyze the first step. *MIPS3* is anti-correlated to *m/z* 333.0592, so this pathway is supposedly less active under stresses and does not contribute to myo-inositol accumulation. In drought stress however, *MIPS1* and *MIPS2* are up-regulated and glucose-6-phosphate is depleted whereas glucose levels go up, in agreement with a hypothetical GroPIns-independent myo-inositol production (Additional file 1: Figure S13; Additional file 2).

In summary, the data support the potential existence of both mammalian pathways from GroPIns to myo-inositol in *Arabidopsis*, but only the putative GDE1-like pathway seems to be activated in stress response. The accumulation of GroPIns under stress may influence transcriptional regulation. Here we discussed putative degradation pathways of GroPIns. It is unclear whether biosynthesis genes also contribute to the accumulation of putative GroPIns in stress. There is no close correlation between *m/z* 333.0592 and the phospholipase A genes annotated in TAIR [4]; the known GroPIns biosynthetic enzyme in mammals belongs to this gene family.

## Conclusions

Analyzing a large dataset along different axes, the study addressed effects of genotypes, stress combination and air humidity as well as relationships between transcriptomic and metabolomic responses in *Arabidopsis thaliana* leaves. While the molecular response to drought and heat stresses surprisingly was not affected by the loss of major aquaporin water channels with prior association to abiotic stress responses [12, 13, 29, 31], drought and heat induced shared and unique response features both at the transcriptomic and at the metabolomic level; drought response involved more differential regulation than heat response. Most of the drought or heat regulations were also present during combined drought-heat stress, where the drought response consequently dominated the heat response. Beyond that, a substantial fraction of molecular drought and heat responses influenced each other negatively or positively, resulting in reduced or enhanced response effects in combined stress, which were functionally explored to give hints whether the strengthening of specific biological processes could improve stress tolerance in all stress scenarios, only in specific stresses or only in certain combined stress situations. Although the extent and nature of stress responses varies with duration and severity of drought and heat conditions, the findings are of interest for further stress interaction studies, including susceptibility to pathogen attack during different abiotic stress situations [2, 36].

On the other hand, already a single stress trigger can evoke a combination of stress factors, reflected by a heterogeneous molecular response and modulated by other climatic factors. An explicit variation of these external factors makes it possible to identify contributions of different stress components to the plant responses. For instance, investigating heat stress with and without air humidity supplement suggested that increased vapor pressure deficit is an important component of heat stress that can be separated from a solely temperature-related component. In our case it appeared to be the primary trigger of metabolomic response, whereas the transcriptomic response was clearly dominated by temperature effects focusing on protein folding. This could mean that ensuring correct protein folding during temperature avoids major disturbances or reorganization of the metabolism. In combined stress, our evidence indicated a larger drought effect and a smaller temperature effect than for heat stress, resulting in parallel effects between the transcriptome and the metabolome, with a reduced response for higher air humidity.

In addition to global or knowledge-based coherency checks between transcriptomic and metabolomic features, the measurements collected in this study allow to explore transcriptome-metabolome correlations across multiple stress conditions with distinct responses in a purely data-driven manner. Certainly, transcriptomic and metabolomic stress responses can differ in their dynamics [84], therefore a single sampling time point per condition cannot capture all dependencies between transcriptome and metabolome in detail. Nevertheless, the case studies presented in this paper show that stress-associated correlated features and combined transcriptome-metabolome biomarkers could be successfully identified. Beyond that, the predictions derived from our correlation networks assist in clarifying functional complementarity and putative stress response roles of known compounds and genes (such as sucrose, cell wall invertases and invertase inhibitors) and in hypothesizing potential functions or metabolic pathways of uncharacterized metabolite compounds, which constitute starting points for future research. Moreover, integrating comprehensive gene expression and non-targeted metabolomics measurements across multiple single and combined abiotic stress conditions as well as selected targeted metabolomics measurements, the dataset produced in this study may serve as a resource to query candidate genes or metabolic features before investigating specific response mechanisms, with the ultimate goal to sustain crop production in variable climate conditions.

## Methods

### Plant materials

The study was performed with wild-type plants and insertion lines from the *Arabidopsis thaliana* ecotype Columbia (Col-0). Wild-type Columbia seeds were originally obtained in the 1990s from George Redei (Col-0), and thereafter propagated in the research group of Anton Schäffner. The loss-of-function mutants *pip2;1–2* (SM_3_35928), *pip2;2–3* (SAIL_169A03) and *pip2;4–1* (SM_3_20853) [85–87] were obtained from the Nottingham *Arabidopsis* Stock Center [88] and had been characterized previously [29, 30]. The double mutant *pip2;1 pip2;2* and the triple mutant *pip2;1 pip2;2 pip2;4* used in this study were generated by crossing the above-mentioned single mutants.

### Plant growth and environmental stress conditions

The plants were grown on soil (peat moss-based *Floragard B seed* (Floragard Vertriebs GmbH, Oldenburg, Germany) mixed with quartz sand (0.6–1.2 mm grit size; Gebrüder Dorfner GmbH, Hirschau, Germany) at a ratio of 8:1) in cultivation trays (PL 2838/48, Pöppelmann, Lohne, Germany) with individual pot size of 4 × 4 cm inner dimensions and 66 ml volume. The cultivation plates were subdivided into aggregates combining six (arranged as 2 × 3) pots. Each six-pack was filled with the same amount of substrate by weight. Later weighting was used to monitor uniform watering or to assess the water content of the soil (see below). One plant was grown per pot. Plants were raised in two 4 × 4 m, walk-in climate simulation chambers with 11 h/13 h light/dark cycle, 200 μmol m^−2^ s^−1^ photosynthetic photon flux density, 22 °C air temperature and 0.79 kPa vapor pressure deficit (VPD), corresponding to 70% relative air humidity. Plants were regularly watered by an automatic flooding system raising the water level up to 60% of the pot height for 15 min; water was completely drained afterwards. For drought (drought (D) and combined drought and heat stresses (DH)) three-week-old plants were finally flooded with water. After one week without watering the soil water content dropped to approximately 30% of the initial water content constituting drought conditions. Control samples had a relative water content of approximately 70% at harvest. Heat stress (H) was applied to both well-watered plants and drought-treated plants, raising the temperature to 33 °C for 6 h from 11:00 a.m. to 5:00 p.m.. For one set of plants, the absolute air humidity was kept unchanged during the temperature increase, resulting in 37% relative air humidity and 3.17 kPa VPD; this condition is labeled “LrH” (low relative air humidity). For another set of plants, the heat treatments were done with supplemented air humidity to maintain the VPD at 0.79 kPa (at 84% relative air humidity); the condition is labeled “HrH” (high relative air humidity).

Five replicates of each genotype were generated for each environmental scenario; they were randomly distributed in the chambers to exclude position effects. Each replicate consisted of seven or eight rosettes that were harvested after treatment, collected into plastic bags (4 oz. 118 mL, Whirl-Pak sampling bags, Sigma-Aldrich, Germany), immediately frozen in liquid nitrogen and stored at −80 °C until use. Control samples were harvested at the same time as treated samples. All samples were collected at 5 p.m. within 15 min. For microarray and FT-ICR-MS analyses, samples were ground at 2500 rpm for 2.5 min using the mixer mill MM 400 (Retsch, Haan, Germany), and aliquots of 100 mg powder were used for RNA or metabolite extraction, respectively.

### Supportive physiological measurements and meta data

Transpiration of four-week-old plants grown on soil was measured using a GFS-3000 portable gas-exchange system fitted with a special cuvette for *Arabidopsis* 3010-A (Walz, Effeltrich, Germany). The air flow to the cuvette was set at 700 mmol s^−1^. During the measurements, the absolute CO_2_ concentration, cuvette temperature, and light intensity in the cuvette were set at 390 ppm, 23 °C and 350 μmol m^−2^ s^−1^ photosynthetic photon flux density, respectively. The relative air humidity was progressively changed to 20%, 40%, 60% and 75%. The transpiration rate of the rosettes was recorded every 30 s for a total time of 8 min and the values of the last 3 min at each relative humidity setting were averaged (Additional file 1: Figure S11).

To assess the water content of rosette leaves upon harvesting after the heat and drought-heat scenarios, a defined amount of fresh material ground in liquid nitrogen was completely dried. The water content was calculated as difference of fresh weight minus dry weight (Additional file 1: Figure S5). The water content of fully watered and drained substrate was regarded as saturated. After complete drying in an oven the residual weight was 35%. These two extremes defined the water content of our substrate as 100% (fully watered, highest weight) to 0% (completely dried, 35% of the starting weight). During growth and in control conditions, approximately 70% water content was maintained by regular watering. Drought treatment reduced the water content to approximately 30% during a week after stopping the regular watering. To monitor the soil evaporation rate under the different heat stress scenarios, six-packs filled with soil/sand mixture were prepared as indicated above, and placed in climate chambers.

### Microarray analysis

Total RNA was extracted using the RNeasy plant mini kit (Qiagen, Hilden, Germany). RNA quality and quantity was checked with an Agilent Bioanalyzer 2100 (Agilent Technologies, Waldbronn, Germany) and a Nanodrop ND-1000 spectrophotometer (Kisker-Biotech, Steinfurt, Germany). Transcriptomic analysis was performed using Agilent At8**✕**60K one-color microarrays (Design ID: 29132, A-GEOD-16892) according to the manufacturer’s instructions. After 17 h hybridization at 65 °C and washing, slides were scanned using the Agilent Microarray Scanner, and data were extracted using the Agilent Feature Extraction Software with the template GE1_1010_Sep10. The preprocessing including background correction, quantile normalization, log_2_ transformation and averaging across probes of the same gene was done with the Bioconductor 2.13 software package limma, version 3.18.13 [27, 28]. Batch effects of the three independent experimental rounds were corrected using the nlme package in R, version 3.1–115 [89]. One experimental round included all six conditions, one included five conditions (without D) and one included four conditions (without LrH conditions). For each genotype, three out of five replicates were selected for microarray analysis (FT-ICR-MS measurements were done with all five replicates, see below). In summary, this yielded 135 microarray samples. The transcriptional data related to the genes *PIP2;1*, *PIP2;2* and *PIP2;4* (AT3G53420, AT2G37170 and AT5G60660), which were eliminated in the loss-of-function mutants, were excluded from further analysis. Apart from that, all genes and arrays from the measurement were kept in the analysis.

The quality of the microarray-based transcriptome analyses was further assessed. There is a good correlation between biological replicates from all rounds, with an average of 0.9916 across all biological groups (Additional file 1: Figure S15). The arrays cluster according to abiotic stress conditions (Additional file 1: Figure S16; Fig. 1a). To independently verify individual results from the microarray analyses, JA- and ABA-responsive genes were assessed by RT-qPCR. One μg of isolated total RNA was reverse transcribed using a QuantiTect Reverse Transcription Kit (Qiagen, Hilgen Germany) according to the manufacturer’s instructions. Gene-specific primer pairs were designed using the Primer Express 3.0 software. Primer pairs are listed in the supplemental material (Additional file 1: Table S3). All primer pairs were evaluated for amplification specificity and efficiency [90]. The qPCR was performed using SYBR Green Sensimix (Bioline, Luckenwalde, Germany) on a 7500 real-time PCR system (Applied Biosystems). Individual PCR reaction mixtures contained 4 μL of diluted cDNA, 10 μL of SYBR Green, and 250 nM of each primer in a final volume of 20 μL. The expression levels of target genes were normalized with the abundance of the constitutive *UBQ5* and *S16* genes [91]. The RT-qPCR experiments were performed with two technical replicates per each biologically independent sample.

### FT-ICR-MS analysis

Metabolite extraction was performed as described previously [92] with slight modifications. Forty-four μg/mL loganin were added to the extraction buffer 1 (methanol/chloroform/H_2_O 2.5:1:1 *v*/v/v) as an internal standard for calibration. Two mL pre-cooled extraction buffer 1 (−20 °C) was added to 100 mg plant material and mixed at 4 °C for 30 min. After centrifugation (10 min, 14,000 rpm, 4 °C), 1 mL of the supernatant was transferred into a fresh 2 mL Eppendorf tube and the remaining pellet was extracted in a second step with 1 mL pre-cooled (4 °C) methanol/chloroform (1:1 *v*/v). After a second centrifugation, 500 μL were taken off and both supernatants were combined. The extract was divided into several 200 μL aliquots and dried completely using a Speed-Vac. For MS analysis, the pellet was redissolved in 200 μl 70% methanol and diluted 1:100 in 70% methanol containing 35 pmol/mL di-alanine as another internal standard and for monitoring the ionization.

A Solarix FT-ICR mass spectrometer (Bruker Daltonics, Bremen, Germany) coupled to a 12 Tesla magnet (Magnex, UK) was used for the experimental study. All ion excitations were performed in broadband mode (frequency sweep radial ion excitation). 300 scans were accumulated for each mass spectrum. Ions were accumulated in the collision cell for 300 ms for thermalization and enrichment prior to ICR ion detection. The instrumental mass range *m/z* 147–1000 amu was scanned. The electrospray ionization source (Apollo II, Bruker Daltonics, Bremen, Germany) was used in the negative ionization mode to ionize the studied analytes in 50% methanolic solution (Lichrosolv, Sigma-Aldrich, Schnelldorf, Germany). The sample solutions were injected directly to the ionization source by the use of a microliter pump at a flow rate of 2 μL/min. A source heater temperature of 200 °C was maintained and no nozzle-skimmer fragmentation was performed in the ionization source. The instrument was previously calibrated by the use of arginine negative cluster ions starting from a methanolic arginine solution of 5 mg/L.

The measurements were performed for all five replicates of each genotype from the two experimental rounds with four and five conditions, respectively, described in the microarray analysis section. This yielded 135 FT-ICR-MS samples in total. Mass calibration was based on two internal standards (see above) and 18 endogenous metabolites (Additional file 1: Table S4). After quality control of calibrated spectra, two subsequent measurement batches and an outlier were removed and 103 samples were left, for which we performed ^13^C isotope filtering, retaining only peaks where a corresponding isotope peak was detected. Then the noise-cleaned spectra were merged across samples with an error tolerance of 1 ppm. The intensities in each sample were normalized by total ion current. To focus on reliably detected masses, we selected the 663 masses detected in at least two thirds of the samples in at least one condition. The missing values were set to the lower detection threshold (5e+05) and the data were log_2_ transformed. Finally, the data were corrected for experimental round effects and replicate measurement order using the nlme package in R [89]. Mapping of masses to metabolites was performed with MassTRIX [34] and with ChemSpider [72] using the metabolism data sources ChEMBL, BioCyc, AraCyc, MassBank, KEGG and Golm Metabolome Database and a maximum deviation of 2 ppm; matching results were ranked according to increasing monoisotopic mass distance.

### GC-MS analysis

GC-TOF-MS measurements were done at the Faculty of Biology of Ludwig-Maximilians-Universität München (Martin Lehmann). The metabolites were extracted from 100 mg frozen plant material by adding 900 μL 80% methanol (−20 °C) and heating to 70 °C for 15 min, and derivatized for GC-MS analysis [93–95]. Samples were injected into a GC-TOF-MS system (Pegasus HT, Leco, St Joseph, USA). The transfer line, connecting the GC and the TOF-MS, was set to 250 °C, as well as the ion source where the metabolites got ionized and fractionated by an electron pulse of 70 eV. Mass spectra were recorded at 20 scans per second with an *m/z* 35–800 scanning range. Chromatograms and mass spectra were evaluated using ChromaTOF 4.5 and TagFinder 4.1 software [96]. Measurements were done for two conditions (D and control), two genotypes (wild-type and *pip2;1 pip2;2* double mutant) and five replicates each in two experimental rounds (one experimental round overlapping with FT-ICR-MS measurements, both rounds overlapping with microarray measurements). The total number of samples is 38 (two D mutant samples were missed in one round). The features include 112 metabolites and 51 unknown analytes. The data were log_2_ transformed and corrected for experimental round effect with the nlme package [89].

### Statistical analysis

The statistical data analysis was performed with R, version 3.0.3. Principal Component Analysis was done using the prcomp function. For Canonical Correlation Analysis, the rcc function of the mixOmics R package version 5.0-1 [23, 61] was applied on the 56 paired samples from microarray and FT-ICR-MS measurements; the regularization parameters were estimated according to Schäfer and Strimmer [97]. Pearson correlation coefficients were computed by cor.test. The GC-MS data shared 23 samples with the microarray data and 18 samples with the FT-ICR-MS data (all three datasets share 11 samples). Network communities were determined by label.propagation.community and visualized by plot.communities from the igraph package, version 0.7.1 [67, 68]; cliques were detected with the maximal.cliques function from the same package. Subnetworks were drawn with Graphviz, version 2.36, and Cytoscape, version 3.1.1. For differential analysis between conditions or genotypes, linear models were fitted on each omics dataset separately (including all the samples) using the Bioconductor 2.13 package limma, version 3.18.13 [27, 28]; the log_2_ fold change threshold was set to 1 and the adjusted *p*-value threshold was set to 0.05 with FDR as the adjustment method. In the combined stress analysis omitting high air humidity scenarios, conditions were represented by two binary factors (drought/non-drought and heat/non-heat) including an interaction term, otherwise by one multi-valued factor. For GO enrichment analysis of gene lists, the function fisher.test was employed and multiple testing correction by FDR was done with p.adjust; the GO annotation was taken from the org.At.tair.db package, version 2.10.1.

## Additional files

Additional file 1: Supplementary figures and tables. The supplementary figures and tables, combined into a single document, support the findings described in this study and are cited at appropriate places in the main text. (PDF 3152 kb)

Additional file 2: Result lists for stress combination analysis. Mass and gene classifications are provided according to the combined stress model depicted in Fig. 2. Classification is carried out with respect to the regulation in drought stress (D), the regulation in heat stress (H) and the drought:heat interaction term (D:H). The resulting groups are named according to Fig. 2; in addition, the table contains three tiny groups with opposite regulation between drought and heat, which are omitted from Fig. 2. For each gene group, functional annotation and enriched GO terms are additionally provided in separate sheets. (XLSX 312 kb)

Additional file 3: Result lists of condition-specific gene expression and metabolomic mass levels estimated by linear modeling across the whole dataset. Tables show mean log_2_ gene expression values and log_2_metabolomic mass levels for each of the six environmental conditions: control, drought (D), heat with low relative air humidity (H_LrH), heat with high relative air humidity (H_HrH), drought stress combined with low air humidity heat stress (DH_LrH) and drought stress combined with high air humidity heat stress (DH_HrH). Values of all individual samples are available in the processed data file deposited at the ArrayExpress and MetaboLights databases, respectively (Availability of data and materials). (XLSX 2264 kb)

Additional file 4: Result lists for air humidity analysis. Mass and gene lists as well as enriched GO terms are given for the Venn groups in Fig. 3a and b, distinguishing low air humidity-specific variables (LrH only), high air humidity-specific variables (HrH only) and overlapping variables (overlap) for the heat stress setting (H) or the combined drought-heat stress setting (DH). Up-regulated and down-regulated subgroups are shown in separate sheets. (XLSX 409 kb)

Additional file 5: Correlations of targeted metabolites from GC-MS dataset used for evaluation. Pearson correlations between GC-MS-measured metabolites and variables of the main study datasets are given. The first sheet contains correlations of the sucrose inversion variables from Fig. 5a. The second sheet contains correlations between GC-MS metabolites and the network genes from Fig. 5b, including metabolite-gene, gene-gene and metabolite-metabolite correlations. The third sheet contains correlations between GC-MS metabolites and FT-ICR-MS mass variables. (XLSX 15496 kb)

Additional file 6: Results lists for network communities of correlated genes and metabolic masses. The graph structure of each network community shown in Fig. 5b and c is given in dot format readable by Graphviz (http://www.graphviz.org), and significantly enriched GO terms for each community are provided as lists. (XLSX 1518 kb)

## Abbreviations

6MSOH: 6-methylsulphinylhexyl glucosinolate
ABA: Abscisic acid
ABF4: ABA-responsive element binding factor 4
ACX1: Acyl-CoA oxidase 1
AHG1: ABA-hypersensitive germination 1
C/VIF: Cell wall/vacuolar inhibitor of fructosidase
CAD: Cinnamyl alcohol dehydrogenase
CCA: Canonical correlation analysis
CCR2: Cinnamoyl CoA reductase
CHI: Chalcone isomerase
CHS: Chalcone synthase
CWINV: Cell wall invertase
CYP79B2: Cytochrome P450, family 79, subfamily B, polypeptide 2
D: Drought stress
DH_HrH: Combined drought-heat stress with high relative air humidity
DH_LrH: Combined drought-heat stress with low relative air humidity
F3H: Flavanone 3-hydroxylase
FDR: False discovery rate
FT-ICR-MS: Fourier transform ion cyclotron resonance mass spectrometry
GC-MS: Gas chromatography mass spectrometry
GDE: GroPIns phosphodiesterase
GDPD: Glycerophosphodiester phosphodiesterase
GO: Gene ontology
GroPIns: Glycerophosphoinositol
H_HrH: Heat stress with high relative air humidity
H_LrH: Heat stress with low relative air humidity
HSF: Heat shock factor
HSP: Heat shock protein
JA: Jasmonic acid
JAM1: JA-associated MYC2-like 1
JAZ: Jasmonate-zim-domain protein
LTI30: Low temperature-induced 30
LTP3: Lipid transfer protein 3
MBF1C: Multiprotein bridging factor 1C
MIPS: Myo-inositol-phosphate synthase
MYB: Myb domain protein
NIP: NOD26-like major intrinsic protein
OST1: Open stomata 1
p.adj: FDR-adjusted *p*-value
PAP1: Production of anthocyanin pigment 1
PC: Principal component
PCA: Principal component analysis
PIP: Plasma membrane intrinsic protein
PLC: Phospholipase C
PLD: Phospholipase D
PP2C: Clade A protein phosphatases type 2C
RD29A: Responsive to desiccation 29A
ROS: Reactive oxygen species
S16: Ribosomal protein S5 domain 2-like superfamily protein
SA: Salicylic acid
SIP: Small and basic intrinsic protein
SnRK2.6: Sucrose nonfermenting 1-related protein kinase 2.6
TIP: Tonoplast intrinsic protein
UBQ5: Ubiquitin 5
VPD: Vapor pressure deficit
