# Supplementary material for: Relationships between drought, heat and air humidity responses revealed by transcriptome-metabolome co-analysis
Source: BMC Plant Biol. 2017 Jul 10;17:120. doi: 10.1186/s12870-017-1062-y (PMC5504741; doi:10.1186/s12870-017-1062-y)
Supplement: Supplementary file 1 — Supplementary figures and tables. The supplementary figures and tables, combined into a single document, support the findings described in this study and are cited at appropriate places in the main text. (PDF 3152 kb) [file 12870_2017_1062_MOESM1_ESM.pdf]

Georgii et al.
Additional file 1

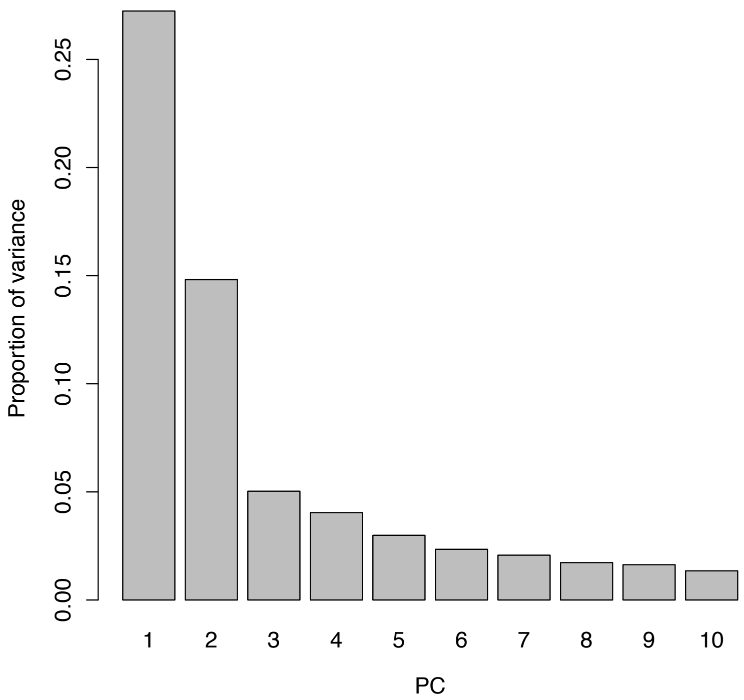

Figure S1. Proportion of variance covered by the first ten principal components of the transcriptomic dataset.

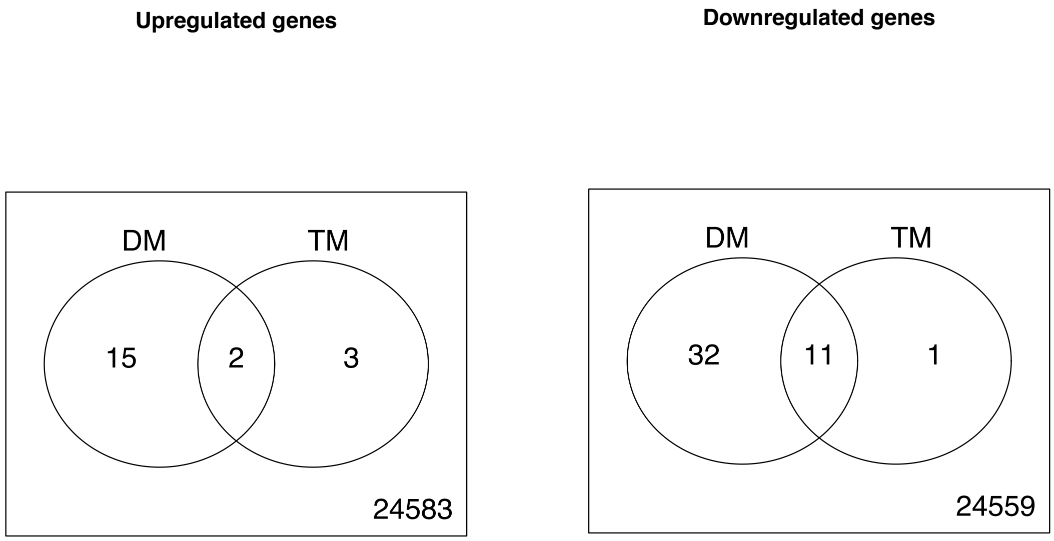

Figure S2. Numbers of differentially regulated genes for aquaporin mutant genotypes vs. wild type under control condition (DM, *pip2;1 pip2;2* double mutant; TM, *pip2;1 pip2;2 pip2;4* triple mutant).

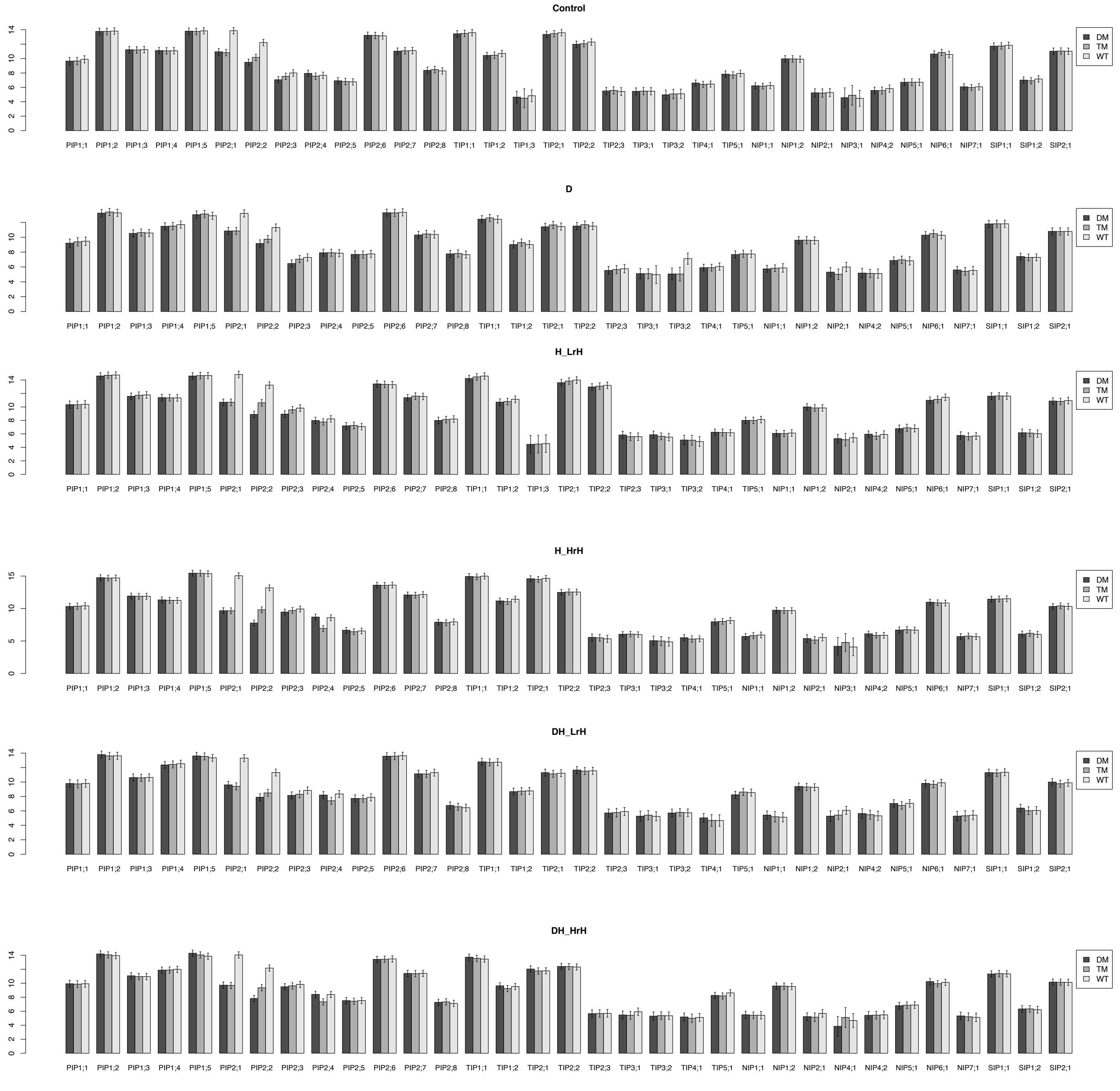

Figure S3. Log_2 leaf expression levels of major intrinsic protein (MIP) genes from microarray analysis, comparing for each environmental condition (indicated in the title of the chart, see Fig. 1 for full names) the following three genotypes: DM, *pip2;1 pip2;2* double mutant; TM, *pip2;1 pip2;2 pip2;4* triple mutant; WT, Col-0 wild type. PIP2;1 and PIP2;2 are the major aquaporins in leaf tissue. The corresponding genes are non-functional in both mutants; in the triple mutant, also the gene encoding PIP2;4 is non-functional. Locus identifiers: PIP1;1: AT3G61430, PIP1;2: AT2G45960, PIP1;3: AT1G01620, PIP1;4: AT4G00430, PIP1;5: AT4G23400, PIP2;1: AT3G53420, PIP2;2: AT2G37170, PIP2;3: AT2G37180, PIP2;4: AT5G60660, PIP2;5: AT3G54820, PIP2;6: AT2G39010, PIP2;7: AT4G35100, PIP2;8: AT2G16850, TIP1;1: AT2G36830,

TIP1;2: AT3G26520, TIP2;1: AT3G16240, TIP2;2: AT4G17340, TIP2;3: AT5G47450, TIP3;1: AT1G73190, TIP3;2: AT1G17810, TIP4;1: AT2G25810, TIP5;1: AT3G47440, NIP1;1: AT4G19030, NIP1;2: AT4G18910, NIP2;1: AT2G34390, NIP4;1: AT5G37810, NIP4;2: AT5G37820, NIP5;1: AT4G10380, NIP6;1: AT1G80760, NIP7;1: AT3G06100, SIP1;1: AT3G04090, SIP1;2: AT5G18290, SIP2;1: AT3G56950.

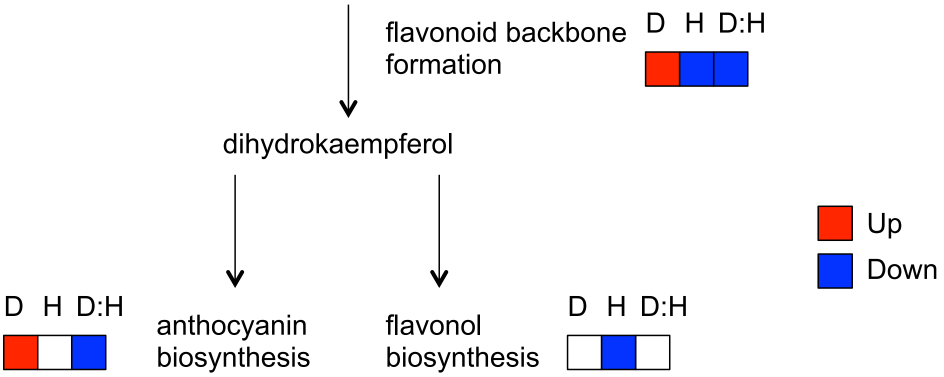

Figure S4. Expression regulation pattern of genes encoding flavonoid backbone formation enzymes, integrating regulation patterns of two separate downstream pathways (D, drought; H, heat; D:H, drought-heat interaction term).

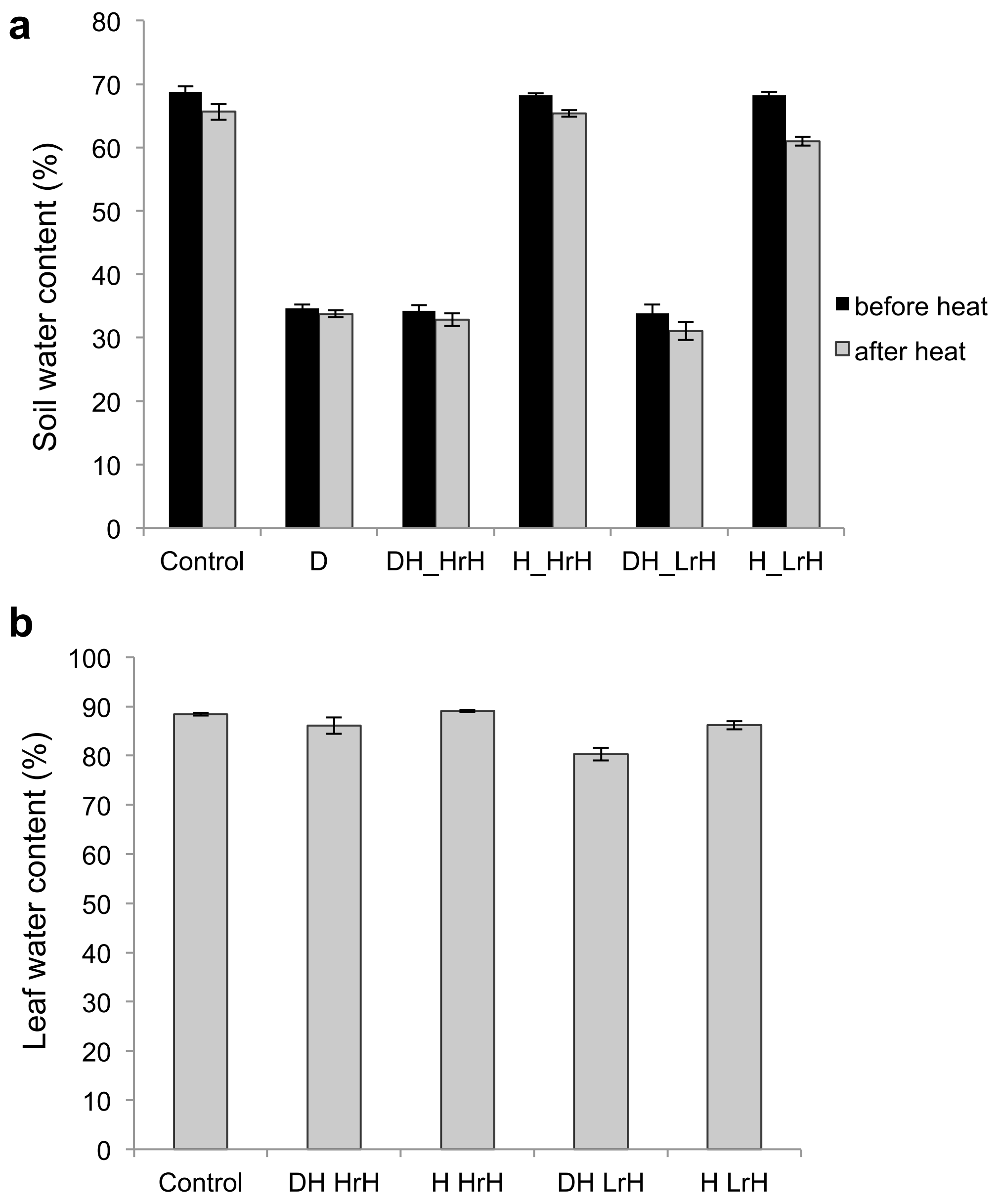

Figure S5. Water content of rosette leaves and soil substrate after heat episodes. (a) Changes in soil water content during a 6-hour heat episode with different air humidity (Methods). To monitor the loss of water from soil during the heat scenarios the weight of the pots from control conditions (approximately 70% water content) and drought treatments (approximately 30% water content) were recorded before and after the heat campaigns. Additional control pots (Control and D (drought)) were kept at ambient control conditions (22 °C, 70% relative air humidity) as a reference. N = 6, ± SD. (b) Leaf water content of rosette leaves upon harvest after the indicated stress treatments (Methods). N = 5, ± SD.

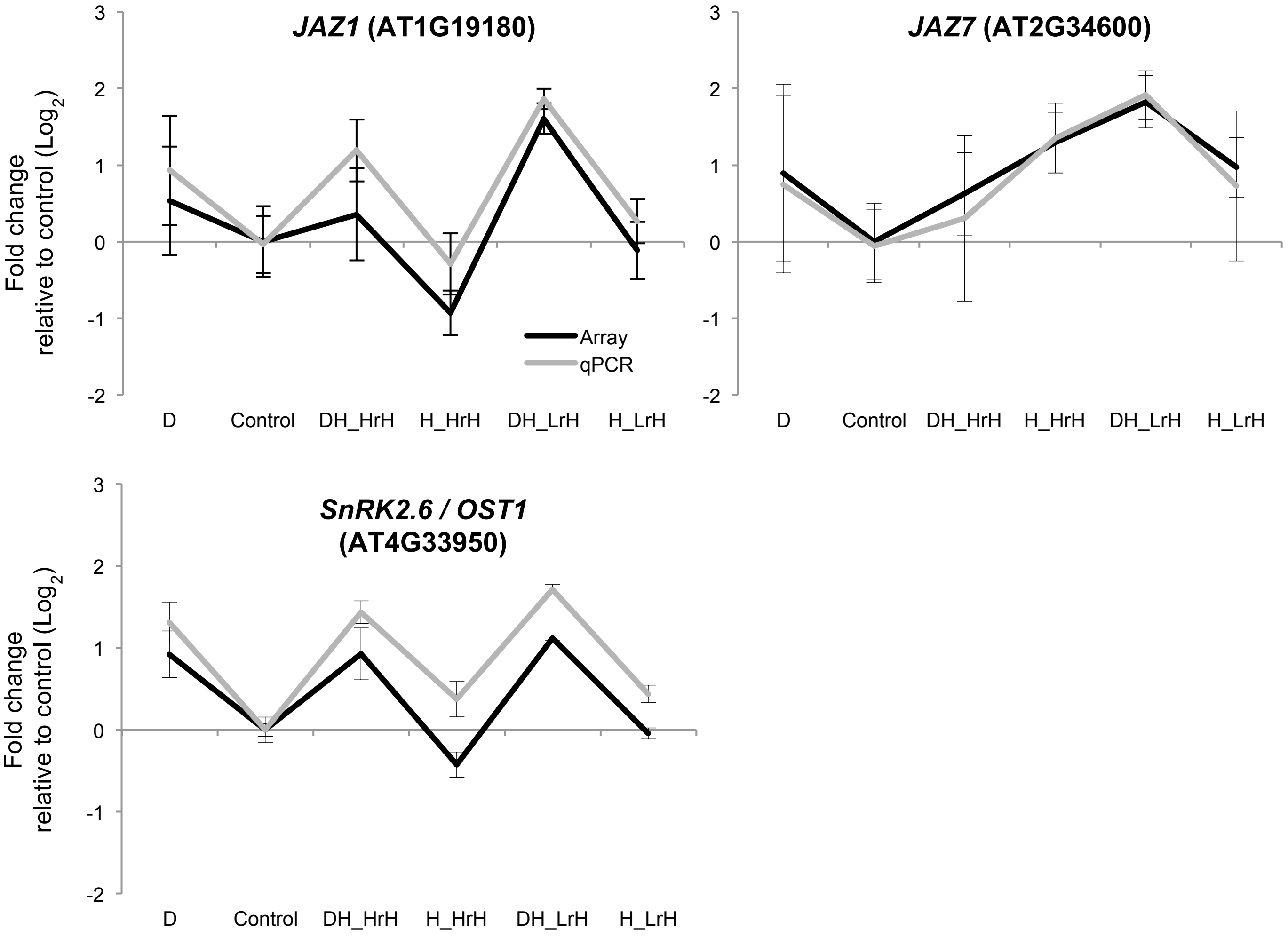

Figure S6. Validation of the microarray results by RT-qPCR. Gene expression of *JAZ1*, *JAZ7* and *SnRK2.6*/*OST1* under stress conditions were determined by RT-qPCR using the same samples as microarray. The expression levels were normalized to *UBQ5* and *S16* transcripts (Methods), and the levels relative to control condition are displayed. Data (mean ± SD) from three independent biological samples of each condition were used for plotting microarray and RT-qPCR analyses.

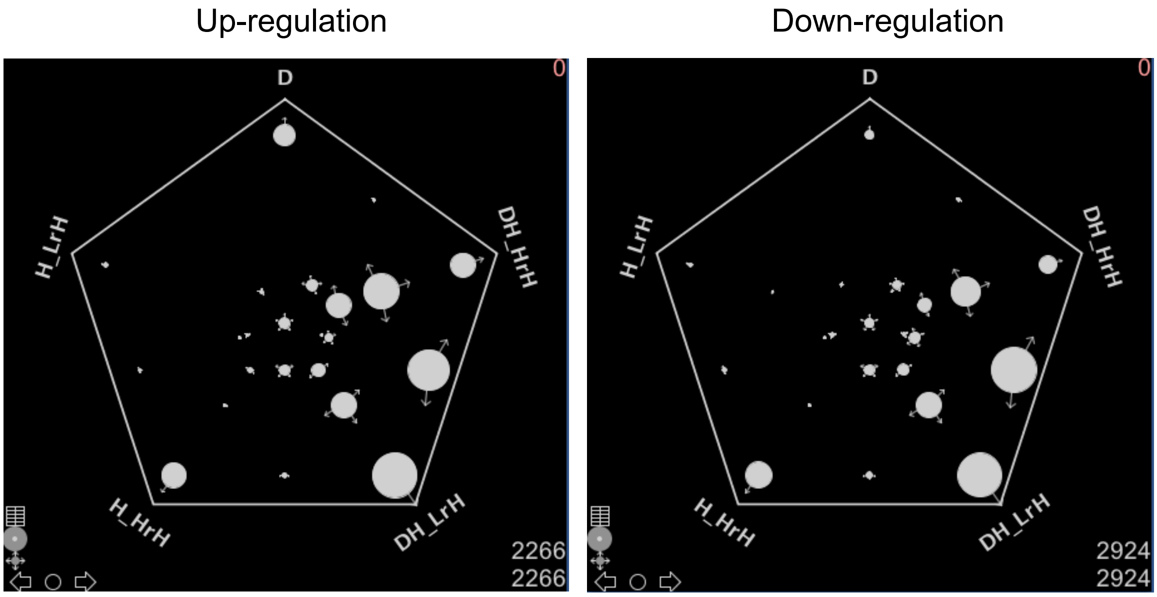

Figure S7. Sungear plot produced with VirtualPlant1.3 (Katari *et al.*, 2010, http://virtualplant.bio.nyu.edu/cgi-bin/vpweb/) to visualize overlaps of differentially regulated genes between different stress conditions. Building on the concept of Venn diagrams, the circles inside the polygon represent the sizes of each possible intersection between up-regulated (left) and down-regulated gene lists (right). Circles are positioned at the average of the corners taking part in the intersection; small arrows around the circles additionally indicate these corners.

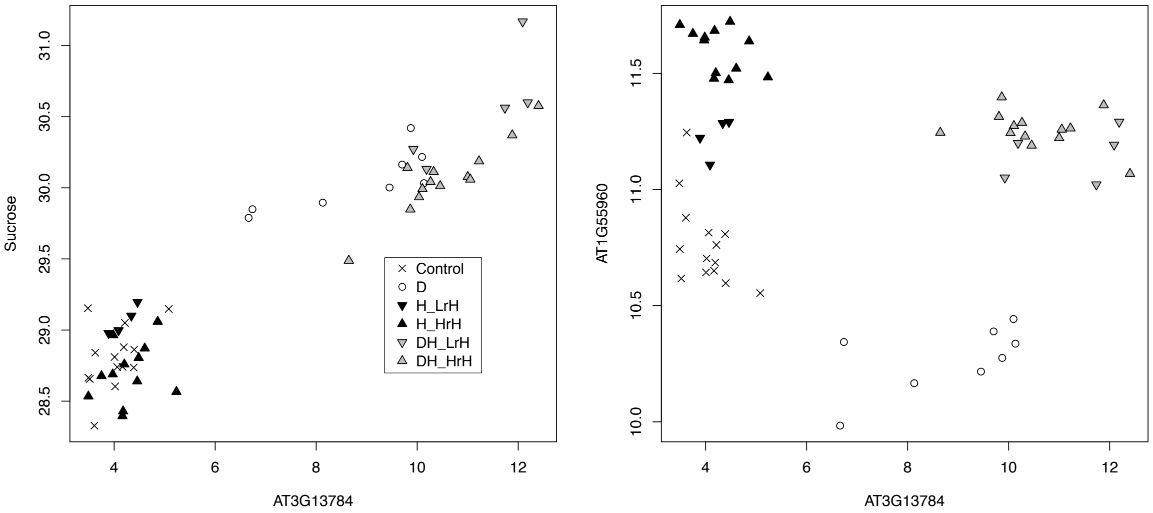

Figure S8. Projection of data onto single variable biomarkers identified from canonical correlation analysis between microarray and FT-ICR-MS data. The left panel shows the top gene (AT3G13784; *CWINV5*) and the top mass (putative sucrose) of the first component. The right panel shows the top genes of the first and second (AT1G55960) component on the horizontal and vertical axis, respectively.

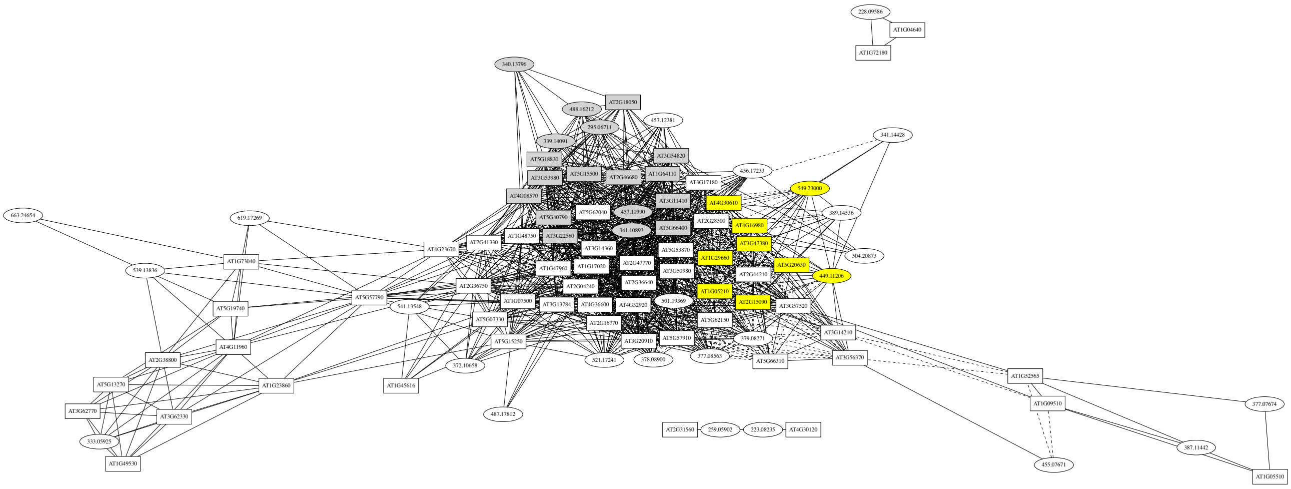

Figure S9. Network of strongest correlations between metabolic masses and genes (solid edges indicate correlation >0.85 with p.adj<10^{-5}, dashed edges correlation < -0.85 with p.adj<10^{-5}). Gray-shaded nodes mark the cliques shown in Figure 5e, yellow-shaded nodes mark the clique whose stress profile is given in Figure S10.

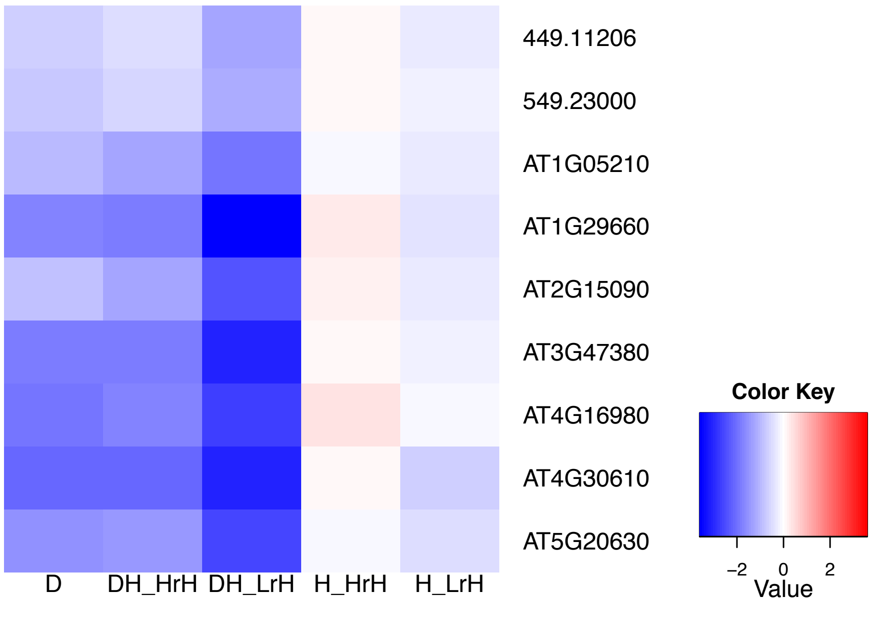

Figure S10. Heatmap of stress response profiles of a fully connected solid-edge subnetwork from Figure S9, see main text for details.

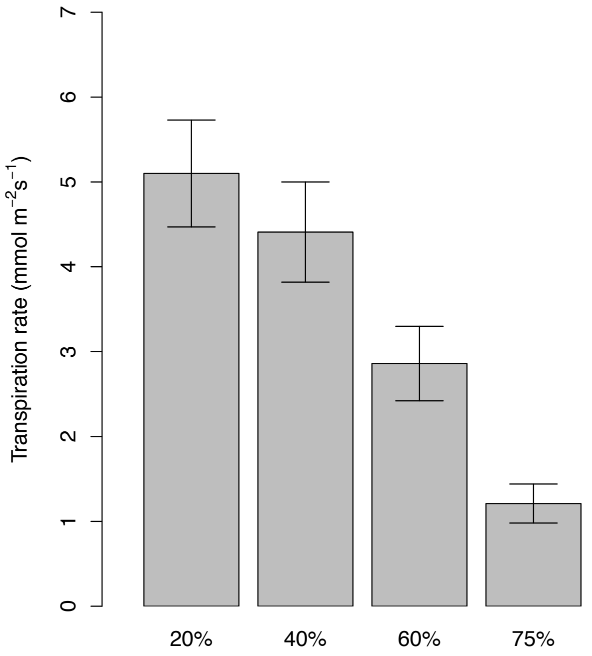

Figure S11. Transpiration rates at different relative air humidity settings. The gas exchange measurements were done with soil-grown four-week-old plants using a portable gas-exchange system fitted with a special cuvette for *Arabidopsis* (Methods). During the measurements, the absolute CO_2 concentration, cuvette temperature, and light intensity in the cuvette were set to 390 ppm, 23 °C and 350 µmol m^{-2} s^{-1} PPFD (photosynthetic photon flux density), respectively. The transpiration rate for each air humidity setting was recorded every 30 s for a total time of 8 min. Average values are taken across the last 3 min. N = 8, ± SD.

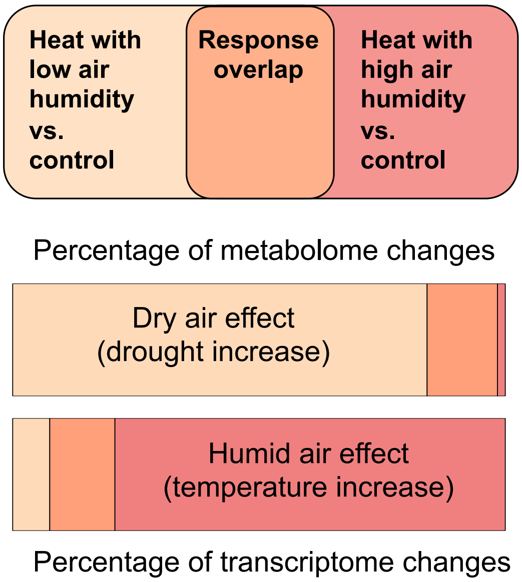

Figure S12. Relative fractions of metabolomic changes (up- or down-regulated features) and transcriptomic changes (up- or down-regulated genes) responding either specifically to heat with low air humidity or specifically to heat with high air humidity or to both. See Fig. 3a for absolute numbers in each partition.

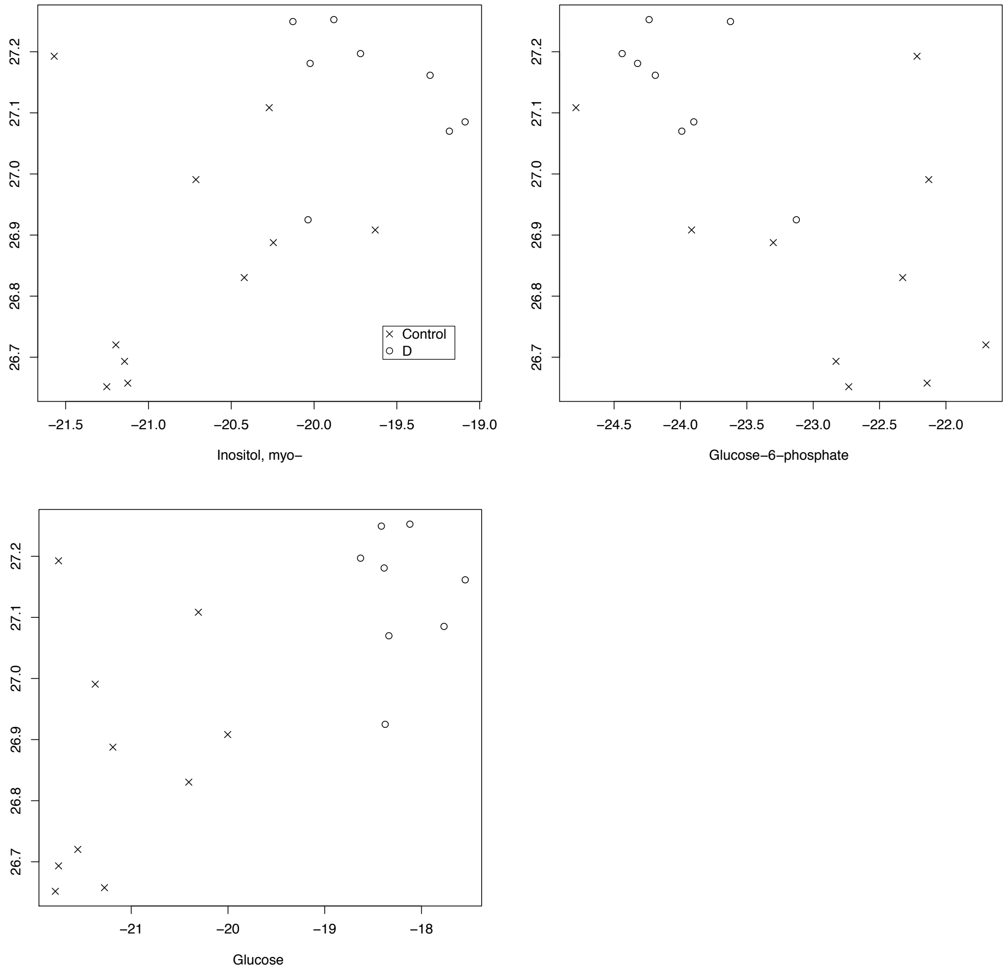

Figure S13. Correlations between mass *m/z* 333.0592 identified by non-targeted FT-ICR-MS analysis (vertical axis), a putative glycerophosphoinositol, and potentially related metabolites quantified by targeted GC-MS measurement (horizontal axis, see label).

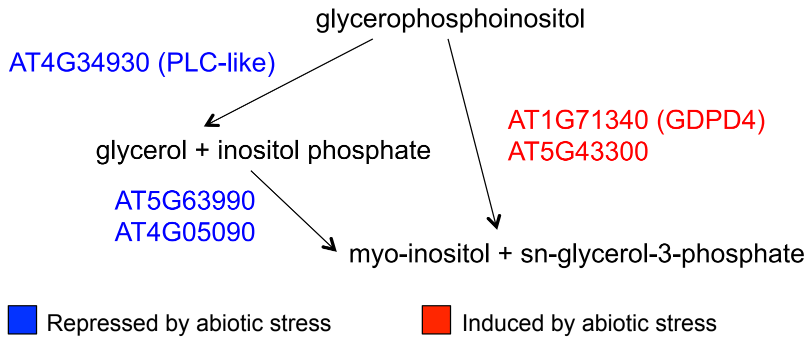

Figure S14. Hypothetical data-derived *Arabidopsis* candidate genes for glycerophosphoinositol pathways known from mammals.

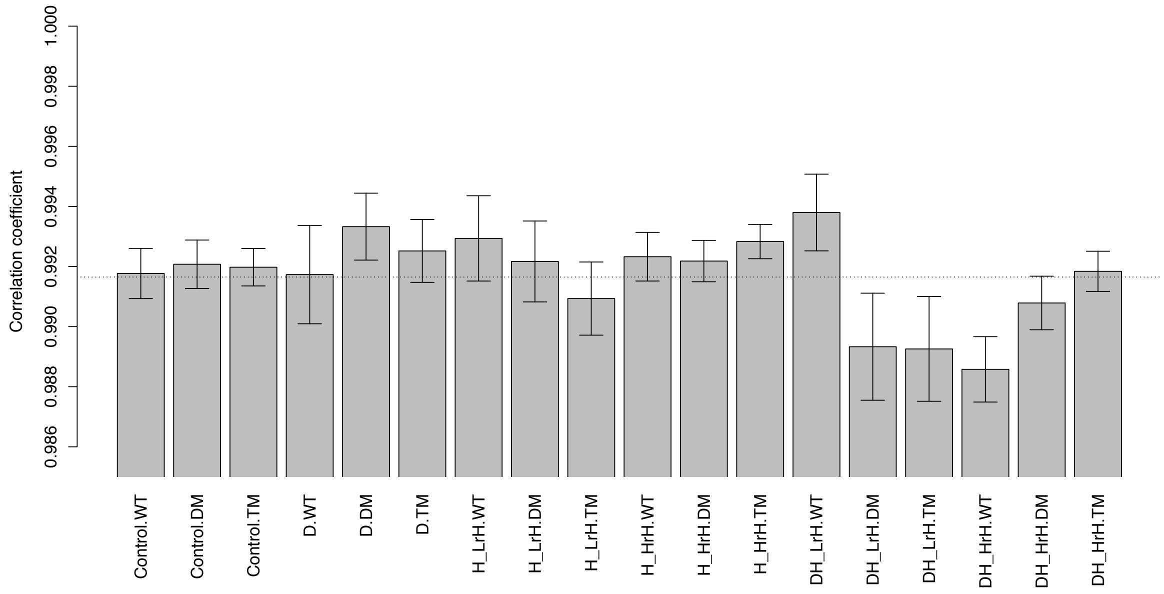

Figure S15. Average Pearson correlation coefficient and standard errors across all pairs of biological replicates from microarray measurements of all experimental rounds. Each biological group is labeled by abiotic stress condition and genotype, separated by a dot (H_HrH: heat with high relative air humidity, H_LrH: heat with low relative air humidity, D: drought, DH_HrH: drought combined with high air humidity heat stress, DH_LrH: drought combined with low air humidity heat stress; WT: wild type, DM: double mutant, TM: triple mutant). The dotted line indicates the overall average pairwise correlation coefficient between biological replicates computed across all biological groups.

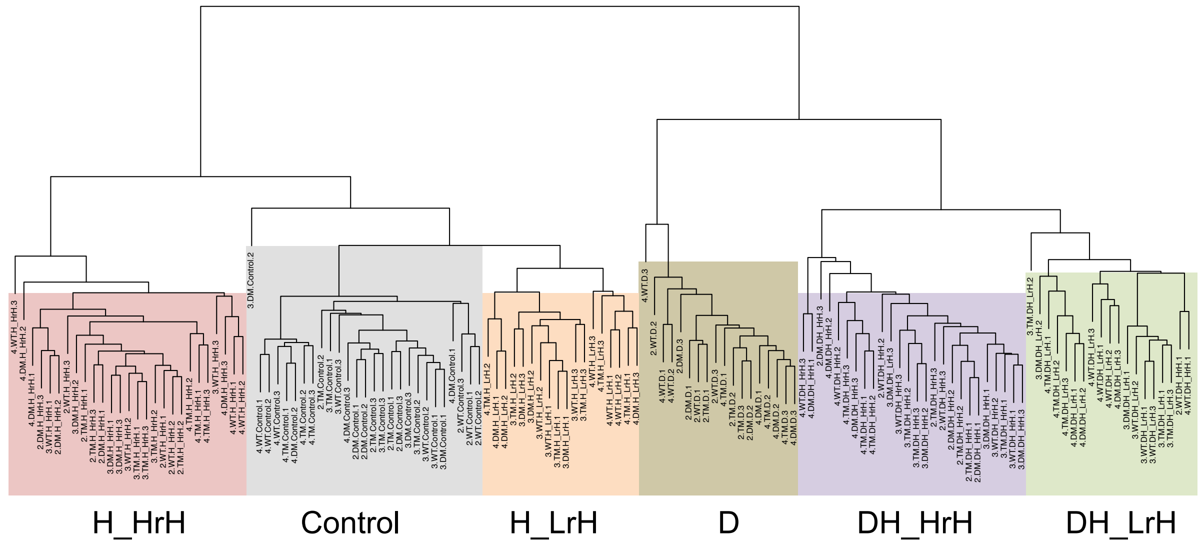

Figure S16. Hierarchical clustering of microarray data. The clustering was done with the function hclust in R with the average linkage method; the distance measure is 1-r, where r is the Pearson correlation coefficient. The arrays cluster according to the abiotic stress conditions (H_HrH: heat with high relative air humidity, H_LrH: heat with low relative air humidity, D: drought, DH_HrH: drought combined with high air humidity heat stress, DH_LrH: drought combined with low air humidity heat stress). Labels of individual experiments indicate separate campaigns, genotype, conditions and biologically independent replicates. See also Fig. 1a.

Table S1. Log_2 expression values of clade A protein phosphatases type 2C across control, single stress and combined stress conditions. Groups are named according to Figure 2 and the classification of responses with respect to drought regulation, heat regulation and the drought:heat interaction term is given (see Additional file 2).

Gene Name	AGI code	Control	D	H_LrH	DH_LrH
**Group 2.1 (1,0,0)**					
PP2CA	AT3G11410	11.723	13.935	12.434	14.569
HAB1	AT1G72770	8.890	11.007	9.850	11.870
HAB2	AT1G17550	10.452	11.478	11.166	12.275
ABI1	AT4G26080	12.998	14.171	13.448	15.026
ABI2	AT5G57050	10.656	12.580	10.983	13.174
**Group 2.2 (1,1,0)**					
HAI1	AT5G59220	7.762	12.861	9.685	14.973
HAI2	AT1G07430	8.810	12.243	9.988	12.900
**Group 3.1 (1,0,1)**					
HAI3	AT2G29380	4.205	5.555	4.166	6.996
**Group 4 (0,0,1)**					
AHG1	AT5G51760	4.213	4.847	4.088	6.842

Table S2. High temperature heat stress experiments from Genevestigator database used for comparison (https://www.genevestigator.com/gv/plant.jsp; July 2015).

Accession number	Condition
AT00120	38°C for 30 min, 1 h and 3 h
AT00500	gradual increase from 22°C to 37.25°C until 20% inhibition of photosynthetic optimum and to 39.6°C until 30% inhibition of photosynthetic optimum
AT00645	40°C for 20 min and 1 h

Table S3. Oligonucleotide primers used for RT-qPCR.

Gene	AGI code	Primer name	sequence
JAZ1	AT1G19180	JAZ1_F	5’-CAATGGAACTTTAGGCAACTCA-3’
		JAZ1_R	5’-AAGCTTGGTTGCCTAGGAAA-3’
JAZ7	AT2G34600	JAZ7_F	5’-TTACCCATCTTGAGGCTAACG-3’
		JAZ7_R	5’-GAGTCGAATTGTTTGGGATTG-3’
OST1	AT4G33950	OST1_F	5’-GGAGAGATTGTGTACGCAATGT-3’
		OST1_R	5’-GCCAACTCAATAGCAAGCAA-3’
UBQ5	AT3G62250	UBQ5_F	5’-GATGGATCTGGAAAGGTTCAG-3’
		UBQ5_R	5’-ATCTACCGCTACAACAGATCAAG-3’
S16	AT2G09990	S16_F	5’-TTTACGCCATCCGTCAGAGTAT-3’
		S16_R	5’-TCTGGTAACGAGAACGAGCAC-3’

Table S4. Mass list used for internal mass calibration of spectra measured in negative ionization mode.

**Compound**	**Formula [M-H]**	***m/z* [M-H]**
Di-alanine	$C_6H_{11}N_2O_3$ -	159.07752
Dehydroascorbate	$C_6H_5O_6$ -	173.0091599
Ascorbate	$C_6H_7O_6$ -	175.0248108
Glucose	$C_6H_{11}O_6$ -	179.056114
2-Methylcitrate	$C_7H_9O_7$ -	205.0353666
Hexadecanoic acid	$C_{16}H_{31}O_2$ -	255.2329409
Octadecanoic acid	$C_{18}H_{35}O_2$ -	283.2642483
SA-Glucoside	$C_{13}H_{15}O_8$ -	299.077244
Glutathione	$C_{10}H_{16}N_3O_6S$ -	306.0765244
Di-alanine Clusterion	$C_{12}H_{23}N_4O_6$ -	319.162308
Ascorbic acid glucoside	$C_{12}H_{17}O_{11}$ -	337.077639
Sucrose	$C_{12}H_{21}O_{11}$ -	341.108939
1-*O*-Sinapoyl-beta-D-glucose	$C_{17}H_{21}O_{10}$ -	385.114024
Loganin	$C_{17}H_{25}O_{10}$ -	389.145324
Glucoraphanin	$C_{12}H_{22}NO_{10}S_3$ -	436.041135
Glucobrassicin	$C_{16}H_{19}N_2O_9S_2$ -	447.053743
Neoglucobrassicin	$C_{17}H_{21}N_2$O10S_2 -	477.064308
Raffinose	$C_{18}H_{31}O_{16}$ -	503.1618002
Kaempferol di-rhamnoside	$C_{27}H_{29}O_{14}$ -	577.156284
Kaempferol glucoside-rhamnoside	$C_{27}H_{29}O_{15}$ -	593.151199
Kaempferol di-glucoside-rhamnoside	$C_{33}H_{39}O_{20}$ -	755.204024
